# The gut microbiome controls reactive astrocytosis during A**β** amyloidosis via propionate-mediated regulation of IL-17

**DOI:** 10.1172/JCI180826

**Published:** 2025-05-13

**Authors:** Sidhanth Chandra, Jelena Popovic, Naveen K. Singhal, Elyse A. Watkins, Hemraj B. Dodiya, Ian Q. Weigle, Miranda A. Salvo, Abhirami Ramakrishnan, Zhangying Chen, Thomas Watson, Aashutosh Shetti, Natalie Piehl, Xiaoqiong Zhang, Leah Cuddy, Katherine R. Sadleir, Steven J. Schwulst, Murali Prakriya, David Gate, Sangram S. Sisodia, Robert Vassar

**Affiliations:** 1 Ken and Ruth Davee Department of Neurology and; 2 Medical Scientist Training Program, Northwestern University Feinberg School of Medicine, Chicago, Illinois, USA.; 3 Department of Neurobiology, University of Chicago, Chicago, Illinois, USA.; 4 Department of Surgery, Division of Trauma and Critical Care,; 5 Abrams Research Center on Neurogenomics,; 6 Department of Pharmacology, and; 7 Mesulam Center for Cognitive Neurology and Alzheimer’s Disease, Northwestern University Feinberg School of Medicine, Chicago, Illinois, USA.

**Keywords:** Immunology, Microbiology, Neuroscience, Alzheimer disease, Cellular immune response

## Abstract

Accumulating evidence implicates the gut microbiome (GMB) in the pathogenesis and progression of Alzheimer’s disease (AD). We recently showed that the GMB regulates reactive astrocytosis and Aβ plaque accumulation in a male APPPS1-21 AD mouse model. Yet, the mechanism(s) by which GMB perturbation alters reactive astrocytosis in a manner that reduces Aβ deposition remain unknown. Here, we performed metabolomics on plasma from mice treated with antibiotics (ABX) and identified a significant increase in plasma propionate, a gut-derived short-chain fatty acid, only in male mice. Administration of sodium propionate reduced reactive astrocytosis and Aβ plaques in APPPS1-21 mice, phenocopying the ABX-induced phenotype. Astrocyte-specific RNA-Seq on ABX- and propionate-treated mice showed reduced expression of proinflammatory and increased expression of neurotrophic genes. Next, we performed flow cytometry experiments, in which we found that ABX and propionate decreased peripheral RAR-related orphan receptor-γ^+^ (Rorγt^+^) CD4^+^ (Th17) cells and IL-17 secretion, which positively correlated with reactive astrocytosis. Last, using an IL-17 mAb to deplete IL-17, we found that propionate reduced reactive astrocytosis and Aβ plaques in an IL-17–dependent manner. Together, these results suggest that gut-derived propionate regulates reactive astrocytosis and Aβ amyloidosis by decreasing peripheral Th17 cells and IL-17 release. Thus, propionate treatment or strategies boosting propionate production may represent novel therapeutic strategies for the treatment of AD.

## Abstract 2

Chandra et al found that the gut microbiome regulates reactive astrocytosis in a mouse model of Alzheimer’s disease through the gut metabolite propionate and peripheral IL-17.

## Introduction

Alzheimer’s disease (AD) is the most common cause of dementia, a decline in cognition sufficient to impair social and occupational functioning (1). AD is characterized pathologically by the accumulation of proteinaceous aggregates of amyloid β (Aβ) and tau as well as neuroinflammation in the form of reactive microgliosis and astrogliosis. Additionally, GWAS implicate innate and adaptive immunity in AD pathogenesis and progression (1). However, the mechanisms governing immunity in AD are still unclear.

Human studies have found that patients with AD have an altered gut microbiome (GMB) composition compared with healthy controls, suggesting that these changes in the GMB may play a role in AD pathogenesis and progression (2). AD mouse studies using antibiotic (ABX) cocktails to perturb the GMB have consistently shown a reduction in Aβ plaques and neuroinflammatory microglia (3–7). While the connection between the GMB and microgliosis has been well documented, the relationship between the GMB and astrocytes in the context of cerebral amyloidosis has not been extensively investigated (8). We recently reported that ABX-mediated GMB perturbation reduces Glial fibrillary acidic protein–positive (GFAP^+^) reactive astrocytes, GFAP^+^ plaque–associated astrocytes (PAAs), and astrocytic C3 expression, while inducing homeostatic astrocytic morphology only in male APPPS1-21 mice (9). Notably, fecal matter transplants from untreated APPPS1-21 male mice into ABX-treated APPPS1-21 mice to restore the GMB results in a reversal of astrocytic phenotypes and a restoration of amyloidosis (9). Furthermore, in the context of microglial depletion, ABX still reduced GFAP^+^-reactive astrocytosis, PAAs, and C3 expression (9). However, there were no changes in astrocyte morphology after ABX treatment when microglia were depleted. These prior results suggest that ABX influence astrocyte phenotypes through both microglial-independent and -dependent mechanisms (9).

Here, we studied whether gut-derived metabolites may mediate the connection between the ABX-induced GMB compositional changes and brain astrocyte changes. We identified a sex-specific increase in the short-chain fatty acid (SCFA) propionate in male APPPS1-21 mice treated with ABX. Similar to ABX treatment, administration of sodium propionate to APPPS1-21 mice reduced reactive astrocytosis and Aβ plaques. Through a combination of single-nucleus RNA-Seq (snRNA-Seq) and astrocyte-specific translating ribosome affinity purification (TRAP) bulk RNA-Seq, we found that ABX and propionate reduced astrocytic neuroinflammatory signaling and T cell recruitment and activation pathways while increasing neurotrophic support pathways. Furthermore, we performed flow cytometry experiments to understand how ABX and propionate influenced peripheral immunity. We demonstrated reductions in peripheral RAR-related orphan receptor-γ^+^ (Rorγt^+^) CD4^+^ (Th17) cells and IL-17 secretion, which were highly correlated with reactive astrocytosis. Last, using an IL-17 mAb to deplete IL-17, we found that propionate reduced reactive astrocytosis and Aβ plaques in an IL-17–dependent manner. Collectively, our findings suggest that gut-derived propionate may serve as an endogenous protective metabolite against reactive astrocytosis in AD. Exogenous propionate supplementation or strategies to increase endogenous propionate production may represent novel therapeutic strategies for AD.

## Results

### ABX-mediated GBM perturbation reduces GFAP^+^ reactive astrocytes and Aβ plaques while increasing synaptic protein levels.

**Figure 1:**
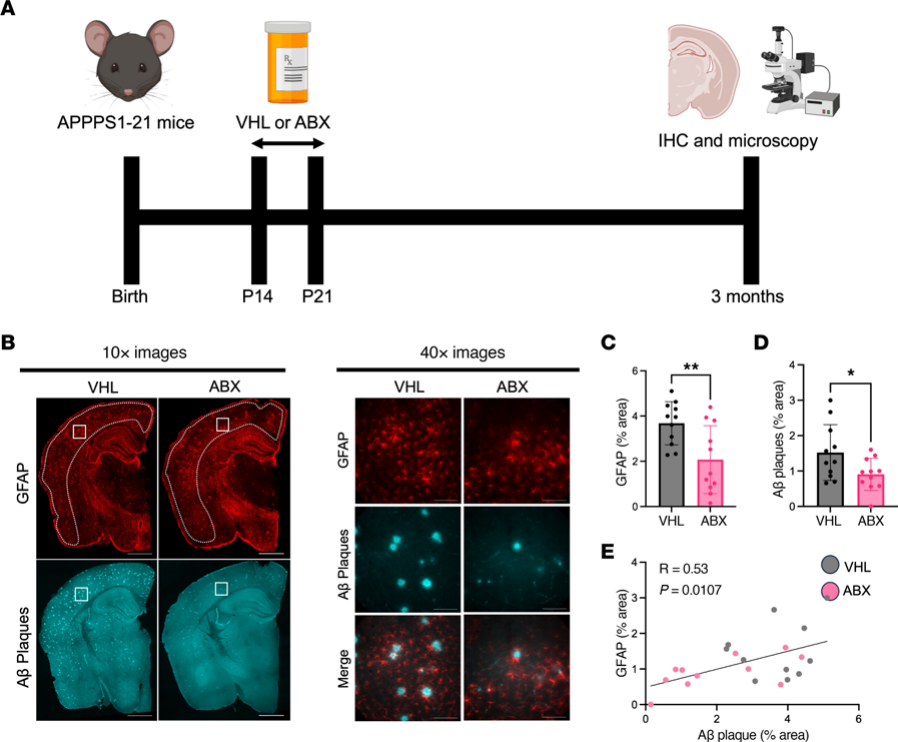
ABX-mediated GBM perturbation reduces GFAP^+^ astrocytes and Aβ plaques in the cortex of APPPS1-21 male mice. (**A**) Schematic depicting the experimental paradigm. (**B**) Representative images of whole brain sections (original magnification, ×10) and high-magnification images of cortex (original magnification, ×40) stained for GFAP^+^ astrocytes and Aβ plaques in APPPS1-21 male mice treated with ABX or VHL control. Quantification of the cortical (**C**) GFAP^+^ astrocyte percentage area and (**D**) the Aβ plaque percentage area in VHL- and ABX-treated APPPS1-21 mice. (**E**) Pearson’s correlation analysis between GFAP^+^ astrocyte percentage area and Aβ plaque percentage area in VHL- and ABX-treated APPPS1-21 mice. Data are expressed as the mean ± SD. *n* = 11/group. Statistics were calculated using a 2-tailed, unpaired Student’s *t* test. *n* = 4 sections per animal. *P ≤ 0.05 and **P ≤ 0.01. Scale bars: 1,000 μm (×10 images) and 100 μm (×40 images). Dotted lines indicate the analyzed area of the cortex.

We previously found that ABX treatment reduces GFAP^+^ astrocytes in male APPPS1-21 mice at 9 weeks and 3 months of age, suggesting that the GMB plays a role in regulating reactive astrocytosis in response to amyloidosis (9). In order to perform astrocyte-specific transcriptional studies, we again administered ABX or water vehicle (VHL) to APPPS1-21 male mice from P14 through P21 (Figure 1A). Similar to our previous report, we found that ABX treatment reduced microbial diversity and increased cecal/body weight (Supplemental Figure 1, A–C; supplemental material available online with this article; https://doi.org/10.1172/JCI180826DS1). Importantly, ABX treatment reduced the cortical GFAP^+^ astrocyte percentage area (Figure 1, B and C), and there was a trend toward a reduction of the area percentage of plaque-associated astrocytes (PAAs) compared with VHL-treated controls at 3 months of age (*P* = 0.0793) (Figure 1B and Supplemental Figure 1D). We also found that ABX reduced Aβ plaques (Figure 1, B and D) and there was a trend toward reduced Aβ plaque size (*P* = 0.0927), similar that reported in previous studies (Supplemental Figure 1E) (3–6, 9). Although we hypothesize that the ABX-mediated reduction in GFAP^+^ reactive astrocytes is not caused by the reduced Aβ plaque pathology, we observed a significant positive correlation between GFAP^+^ reactive astrocytes and Aβ plaques (Figure 1E). This result suggests that higher levels of reactive astrocytes may contribute to Aβ plaque pathology. Similar to our IHC findings, immunoblots of cortical lysates showed a reduction in GFAP levels in ABX-treated APPPS1-21 mice compared with VHL-treated controls (Supplemental Figure 1, F and G). Homeostatic astrocytes are known to have neurotrophic functions, and reactive astrocytes are known to contribute to neurodegeneration (10). With this in mind, we asked whether the reduced reactive astrocytosis associated with ABX treatment occurred concomitantly with increased presynaptic and postsynaptic levels in these mice. Notably, we found an increase in synaptophysin and PSD-95 levels in these mice (Supplemental Figure 1, F, H, and I), suggesting that ABX treatment led to improved synaptic health. Overall, these results suggest that the increased presynaptic and postsynaptic protein levels we observed may have been due to a reduction in reactive astrocytosis and potentially increased astrocytic neurotrophic function.

### Plasma metabolomics reveals a selective increase in the SCFA propionate in ABX-treated male APPPS1-21 mice that correlates negatively with reactive astrocytosis.

**Figure 2:**
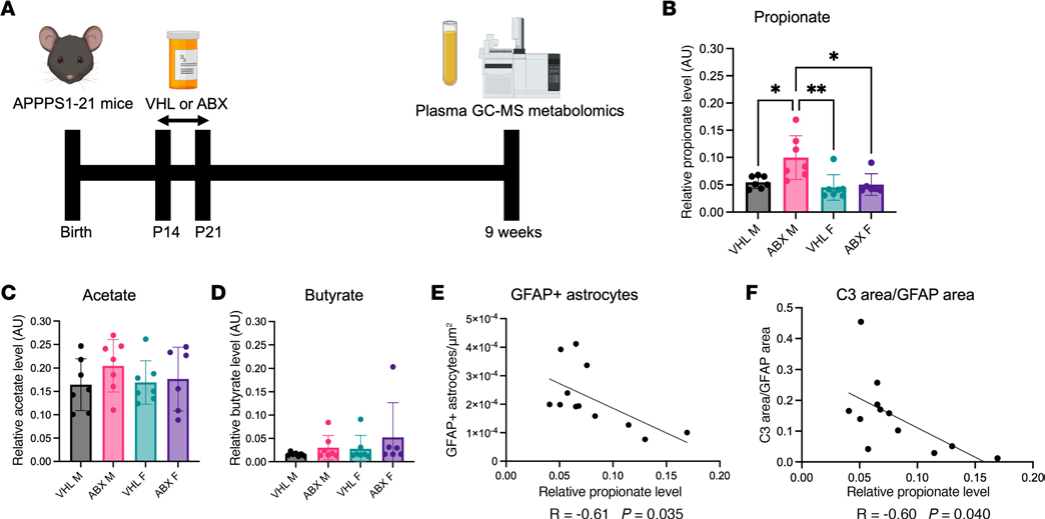
ABX-mediated GBM perturbation increases plasma propionate levels, which negatively correlate with reactive astrocytosis in APPPS1-21 male mice. (**A**) Schematic depicting the experimental paradigm. Relative GC-MS quantification of the SCFAs (**B**) propionate, (**C**) acetate, and (**D**) butyrate in the plasma of VHL- and ABX-treated APPPS1-21 male (M) and female (F) mice. Pearson’s correlation analysis between (**E**) GFAP astrocyte percentage area and (**F**) C3 area/GFAP area and plasma propionate levels in VHL- and ABX-treated male APPPS1-21 mice. Data are expressed as the mean ± SD. *n* = 6–7/group. Statistics were calculated using 2-way ANOVA. *P ≤ 0.05 and **P ≤ 0.01.

Gut microbial metabolism is often perturbed in diseases involving GMB changes (11). In male APPS1-21 mice, we previously observed ABX-induced changes in GMB composition that were associated with changes in astrocytic phenotypes (9). To determine changes in microbial metabolism that may mediate the effects of GMB changes in astrocytes, we used gas chromatography/mass spectrometry (GC-MS) to metabolically profile plasma. Specifically, we analyzed plasma from 9-week-old APPPS1-21 male and female mice that were treated with ABX or VHL control (Figure 2A and Supplemental Figure 2). This metabolic assay was designed for increased sensitivity for GMB-derived metabolites. Surprisingly, we found a sex-specific increase in the SCFA propionate in ABX-treated male APPPS1-21 mice but not female mice and found no significant changes in the levels of the SCFAs acetate or butyrate (Figure 2, B–D). Additionally, we profiled 13 other metabolites in the plasma and found no changes in ABX-treated mice compared with VHL-treated mice (Supplemental Figure 2). The ABX-mediated increase in propionate was particularly interesting because when we previously profiled the GMB composition in these mice, we found that of the 10 genera that were altered by ABX treatment in male mice, only 1 was increased rather than decreased (9). This genus, *Akkermansia*, was significantly increased by ABX treatment only in male APPPS1-21 mice (Supplemental Figure 3A; the *Akkermansia* abundance data for these same ABX-treated mice were obtained from Chandra et al.; ref. 9), which has been documented in previous studies (4). *Akkermansia* is a SCFA-producing bacteria that generates high levels of propionate (12). Importantly, Spearman correlations of *Akkermansia* levels revealed a positive association with propionate levels. This result suggests that the increase in propionate may have been mediated by *Akkermansia* (Supplemental Figure 3B). Propionate levels correlated negatively with measures of reactive astrocytosis, such as GFAP^+^ astrocytes and the complement factor C3 area within GFAP^+^ astrocytes (Figure 2, E and F). Propionate levels correlated positively with homeostatic increases in astrocyte process number and length (trends), which we previously reported to have increased upon ABX treatment in APPPS1-21 male mice (9) (Supplemental Figure 3, C and D). Cumulatively, these results suggest that gut-derived propionate may play a role in an ABX-mediated reduction in reactive astrocytosis and induction of a more homeostatic astrocyte state.

### Exogenous sodium propionate administration reduces GFAP^+^ reactive astrocytosis and amyloidosis.

**Figure 3:**
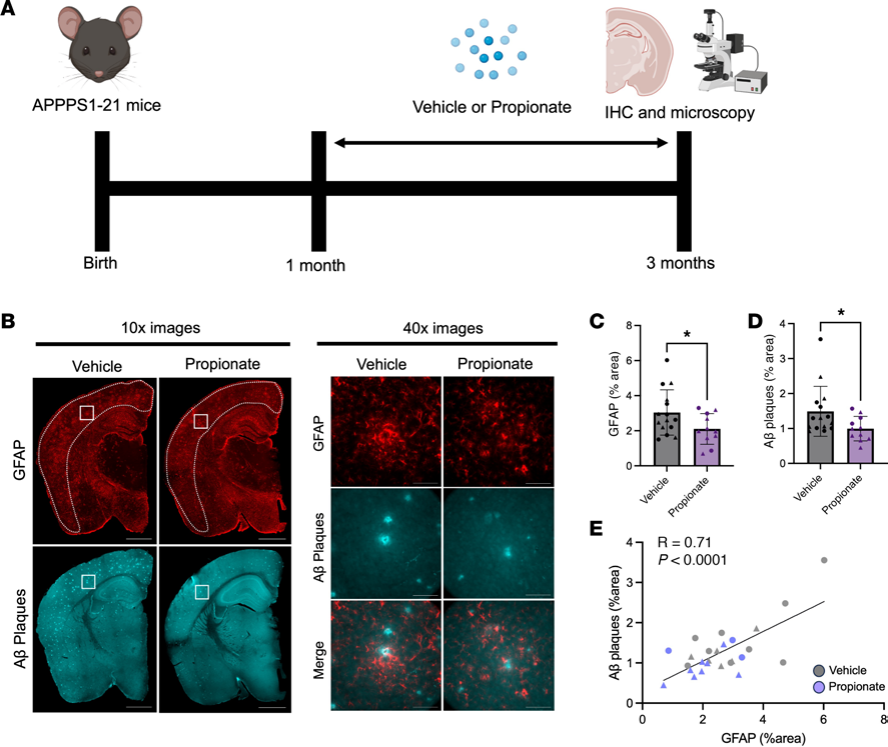
Exogenous propionate treatment reduces GFAP^+^ astrocytes and Aβ plaques in male and female APPPS1-21 mice. (**A**) Schematic depicting the experimental paradigm. (**B**) Representative images of whole brain sections (original magnification, ×10) and cortex (original magnification, ×40) tissue stained for GFAP^+^ astrocytes and Aβ plaques in APPPS1-21 mice treated with VHL control or propionate. Quantification of cortical (**C**) GFAP^+^ astrocyte percentage area and (**D**) Aβ plaque percentage area in VHL- and propionate-treated male and female APPPS1-21 mice. (**E**) Pearson’s correlation analysis between GFAP^+^ astrocyte percentage area and Aβ plaque percentage area in VHL- and propionate-treated male and female APPPS1-21 mice. Data are expressed as the mean ± SD. *n* = 11–15/group. Four sections were used per animal. *P ≤ 0.05, by 2-tailed, unpaired Student’s *t* test. Scale bars: 1,000 μm (original magnification, ×10) and 100 μm (original magnification, ×40). Males are denoted by triangles and females by circles. Dotted lines indicate the cortex area analyzed.

To test whether propionate mediates ABX-induced reductions in GFAP^+^ reactive astrocytosis, we exogenously administered sodium propionate in drinking water of male and female APPPS1-21 mice from month 1 to month 3 (Figure 3A). Importantly, we found that sodium propionate treatment reduced the area occupied by GFAP^+^ astrocytes (Figure 3, B and C), and there was a trend toward a reduction in PAAs (*P* = 0.0554) (Figure 3B and Supplemental Figure 4A). Furthermore, sodium propionate treatment reduced Aβ plaques (Figure 3D), and there was a trend toward a reduction in Aβ plaque size (*P* = 0.1051) (Supplemental Figure 4B). We observed a significant positive correlation between the GFAP^+^ astrocyte area percentage and the Aβ plaque area percentage, suggesting that reactive astrocytes may contribute to Aβ plaque accumulation (Figure 3E). Finally, we profiled the levels of SCFAs including propionate in the brain cortex and found detectable levels in only 1 of 11 propionate-treated samples (Supplemental Figure 4C). This result suggests that the primary action of propionate occurs in the periphery rather than directly in the brain. Additionally, we found no changes in butyrate or acetate levels in the cortex of propionate-treated mice compared with VHL-treated control mice (Supplemental Figure 4, D and E).

### snRNA-Seq reveals a reduction in astrocyte neuroinflammatory and developmental pathways and an increase in neurotrophic subclusters and pathways in APPPS1-21 mice treated with ABX.

**Figure 4:**
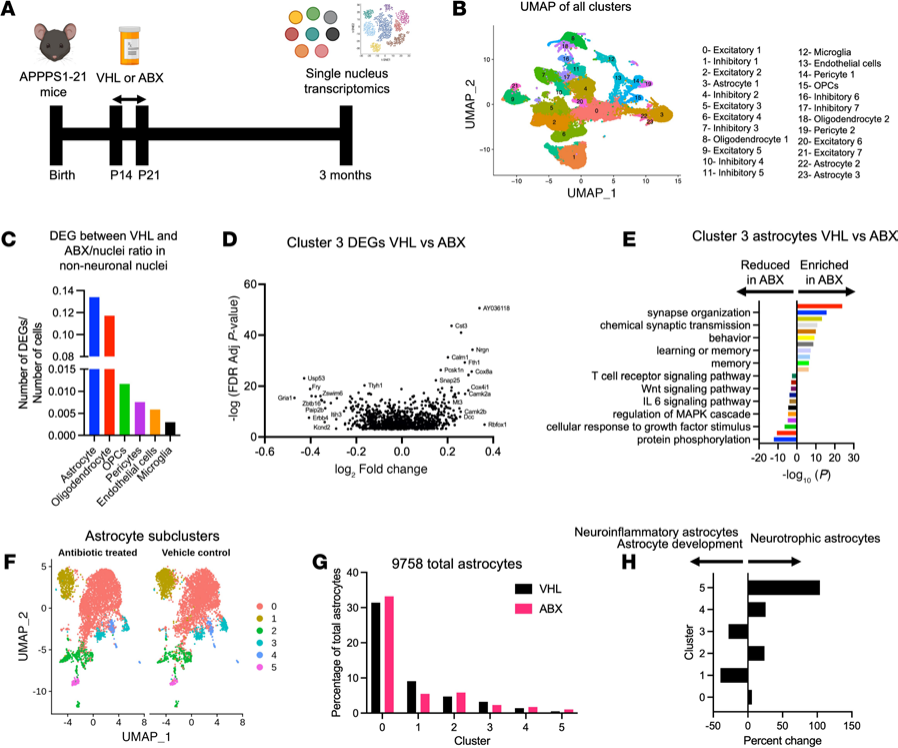
snRNA-Seq reveals changes in astrocytic transcription and subclusters upon ABX-mediated GMB perturbation in APPPS1-21 male mice. (**A**) Schematic depicting the experimental paradigm. (**B**) UMAP plot containing sequenced nuclei from VHL- and ABX-treated APPPS1-21 male mice. (**C**) Number of DEGs per nucleus between VHL- and ABX-treated mouse non-neuronal nuclei. (**D**) Volcano plot of DEGs in cluster 3 astrocytes between VHL- and ABX-treated mice. Adj, adjusted. (**E**) Pathway analysis depicting up and downregulated molecular pathways in cluster 3 astrocytes between VHL- and ABX-treated mice. (**F**) UMAP analysis of clusters 3, 22, and 23 from the UMAP in **B** identified 6 subclusters (subclusters 0–5) of astrocytes in VHL- and ABX-treated APPPS1-21 mice. (**G**) Percentage of astrocyte subclusters in VHL- and ABX-treated mice. (**H**) Percentage change in astrocyte subclusters between VHL- and ABX-treated mice. DEGs and pathways were determined using MAST and Metascape, respectively, with a log_2_ fold change cutoff of 0.25 and an FDR cutoff of 0.001.

To determine how the astrocyte transcriptional state is altered by ABX treatment, we performed snRNA-Seq on nuclei isolated from cortices of APPPS1-21 male mice that were treated with ABX or VHL and sacrificed at 3 months of age. We sequenced 109,581 nuclei in total. Automated clustering using Seurat yielded 23 total clusters, which were manually annotated with classical cell markers (Figure 4, A and B, and Supplemental Figure 5A). Three of the 23 total clusters were annotated as astrocytes (clusters 3, 22, and 23) by their expression of astrocyte-specific genes (Figure 4, A and B, and Supplemental Figure 5B).

Interestingly, astrocytes had the most differentially expressed genes (DEGs) when comparing VHL versus ABX treatment of any non-neuronal nuclei (Figure 4C) (DEGs for VHL vs. ABX per cluster are in Supplemental Table 5). This result suggests that ABX-mediated GMB perturbation has a relatively substantial effect on astrocytic gene expression compared with other cell types. However, it should be noted that astrocytes were among the most numerous cell types in our analysis, so we had more power to detect DEGs compared with other cell types, such as microglia, which had a much smaller representation. Cluster 3 contained the most astrocyte nuclei, and DEG and pathway analysis via Metascape revealed an increase in neurotrophic pathways such as synapse organization, chemical synaptic transmission, behavior, and memory (Figure 4, D and E). Furthermore, we detected a decrease in neuroinflammatory and astrocyte development pathways, such as protein phosphorylation, regulation of the MAPK cascade, IL-6 signaling, WNT signaling, and T cell receptor signaling (Figure 4, D and E). Altogether, these results indicate a phenotypic shift of astrocytes in ABX-treated APPPS1-21 male mice toward a more homeostatic, neurotrophic state.

To get a better understanding of how ABX-induced GMB perturbation alters astrocyte heterogenous subclusters/subtypes, we subclustered all 9,758 astrocyte nuclei in their own computational space (Figure 4F). Automated subclustering revealed a total of 6 subclusters (subclusters 0–5; DEGs for each subcluster are listed in Supplemental Table 1). We found changes in the percentages of astrocytes in several of those subclusters in ABX-treated mice compared with VHL control–treated mice (Figure 4G). Some subclusters showed reduced astrocyte percentages, such as subclusters 1 (40% reduction) and 3 (28% reduction), which seemed to be enriched in the expression of genes involved in both neurodevelopment and neuroinflammation. Subcluster 1 was enriched in glial cell differentiation, gliogenesis, glial cell development, and astrocyte activation (Supplemental Figure 5). Subcluster 3 was enriched in the expression of genes in pathways such as enzyme-linked receptor protein signaling pathways, gliogenesis, glial cell differentiation, glial cell development, and spinal cord injury (Supplemental Figure 5).

Additionally, some subclusters showed increased percentages of astrocytes in ABX-treated mice compared with VHL-treated mice, such as subclusters 2 (24% increase), 4 (26% increase), and 5 (103% increase), which all appeared to be enriched in homeostatic supportive astrocyte functions (Figure 4H and Supplemental Figure 5). Subcluster 2 was enriched in metabolic and neurotrophic pathways, including those for synapse organization, the neuronal system, and regulation of trans-synaptic signaling (Supplemental Figure 5). Subcluster 4 was enriched in cilium movement, cell projection assembly, and microtubule cytoskeletal organization (Supplemental Figure 5). Subcluster 5 was enriched in pathways such as those for synapse organization, the neuronal system, and regulation of trans-synaptic signaling (Supplemental Figure 5). Together, these data suggest that ABX-mediated GMB perturbation reduced astrocytic neuroinflammatory and development subclusters, while increasing neurotrophic astrocyte subclusters.

Of note, the mice used for our snRNA-Seq experiment were housed at the University of Chicago rather than Northwestern University. Because gut microbiota can be influenced by the animal facility location, we profiled the fecal microbiomes of 9-week-old APPPS1-21 mice housed in the 2 facilities using 16S rRNA-Seq. We found there were no changes in α diversity at a phylum level, but there was reduced α diversity at the genus level in mice at the Northwestern facility compared with University of Chicago facility (Supplemental Figure 6, A and B). β-Diversity analysis showed a separation of the microbiomes of mice housed at Northwestern compared with those at the University of Chicago at the phylum and genus levels (Supplemental Figure 6, C and D). The difference in microbial diversity between the facilities was a limitation of this experiment. However, it has been previously shown that, although mouse GMBs between facilities may be different, similar effects on amyloidosis and neuroinflammation are achieved when using the same combination of ABX or other GMB-targeted interventions (3–7, 9, 13).

### TRAP-Seq reveals a decrease in astrocyte neuroinflammatory pathways and an increase and neurotrophic pathways in APPPS1-21 mice treated with ABX or propionate.

**Figure 5:**
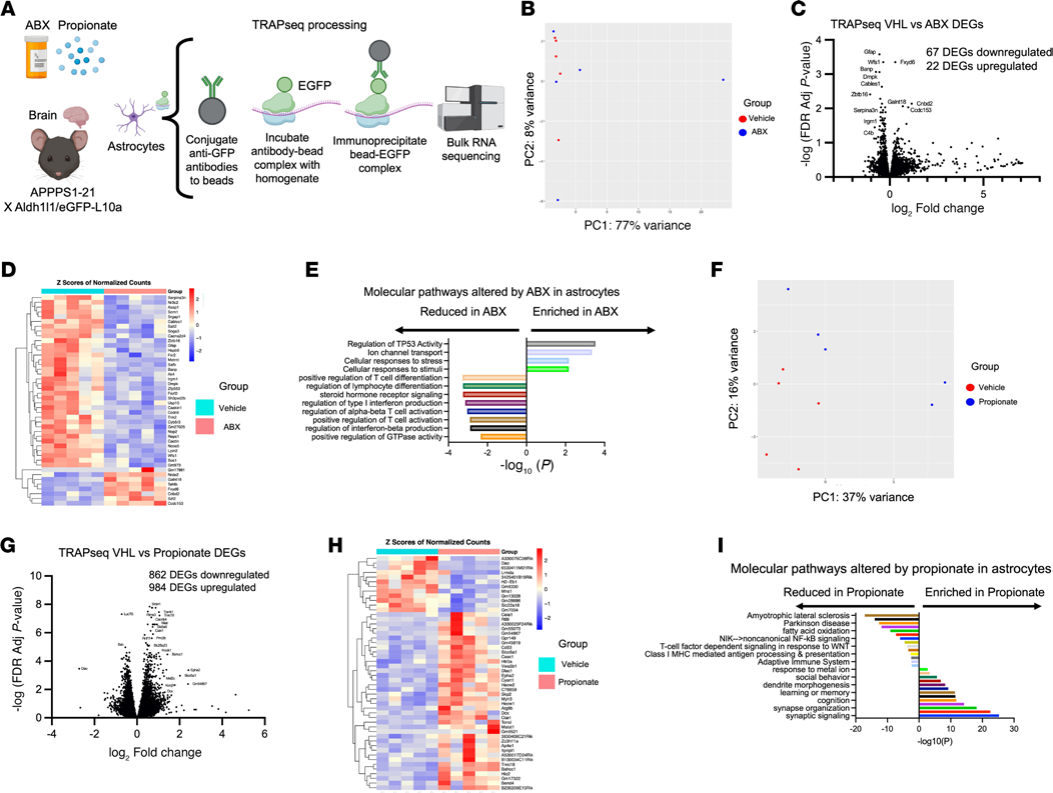
TRAP-Seq reveals changes in astrocytic transcription upon ABX-mediated GMB perturbation and exogenous propionate treatment in APPPS1-21 mice. (**A**) Schematic depicting the experimental paradigm. (**B**) PCA plot of VHL- and ABX-treated TRAP-Seq samples. (**C**) Volcano plot of DEGs in ABX-treated versus VHL-treated APPPS1-21 male mice. (**D**) Heatmap depicting the top upregulated and downregulated DEGs in ABX-treated versus VHL-treated APPPS1-21 male mice. (**E**) Pathway analysis depicting up- and downregulated molecular pathways in astrocytes between VHL- and ABX-treated mice. (**F**) PCA plot of VHL- and propionate-treated TRAP-Seq samples. (**G**) Volcano plot of DEGs in propionate-treated versus VHL-treated APPPS1-21 male mice. (**H**) Heatmap depicting the top upregulated and downregulated DEGs in propionate-treated versus VHL-treated APPPS1-21 male mice. (**I**) Pathway analysis depicting up- and downregulated molecular pathways in astrocytes between VHL- and propionate-treated mice. *n* = 5/group. DEGs were determined using DESeq2 with an FDR cutoff of 0.1. Pathway analysis was conducted using Metascape.

In addition to snRNA-Seq, we profiled the astrocyte transcriptional/translational state using translating ribosome affinity purification (TRAP) bulk RNA sequencing (TRAP-Seq). In TRAP-Seq, a ribosomal protein L10a is fused to EGFP under the control of a cell-type–specific promoter in a transgenic mouse model (14). This allows for bead-based immunoprecipitation of polysomal mRNA from specific cell types. Because this method allows for isolation of polysomal mRNA that is being actively translated, the mRNA content profiled should closely resemble the protein content (14). Using the *Aldh1l1-EGFP/Rpl10a* bacTRAP mouse model, we purified polysomal mRNA from astrocytes and performed bulk RNA-Seq (Figure 5A). We crossed *Aldh1l1-EGFP/Rpl10a* mice with APPPS1-21 mice, treated the mice with ABX, propionate, or VHL, and performed TRAP-Seq (Figure 5A).

Principal component analysis (PCA) showed a separation between VHL and ABX-treated mice, with 77% the of variance being explained by the first PC and 8% of the variance being explained by the second PC (Figure 5B). Of note, we observed a potential outlier in the PCA plot, which could be a limitation of the downstream analysis. We found a total of 89 DEGs in astrocytes of ABX-treated mice compared with VHL-treated mice (Figure 5, C and D, and Supplemental Table 2). Of the 89 DEGs, 67 were downregulated in ABX-treated mice and 22 were upregulated (Figure 5, C and D). Pathway analysis showed that there was in fact a decrease of several proinflammatory pathways such as regulation of IFN-β production, positive regulation of T cell activation, regulation of α-β T cell activation, regulation of type I IFN production, and positive regulation of T cell differentiation (Figure 5E). We also observed increases in a few pathways, such as regulation of TP53 activity, ion channel transport, and cellular responses to stress and stimuli (Figure 5E). We validated ABX-induced astrocytic gene expression changes using quantitative PCR (qPCR) to quantify the expression of a subset of genes identified in our TRAP-Seq experiment: *Gfap*, *Serpina3n*, *Irgm1*, *Bag3*, *Fbxo32*, *Cyp7b1*, and *Fezf2* (Supplemental Figure 7). These genes were chosen because they overlapped with those DEGs in our propionate experiment and for which validated commercial primers were available. As expected, our qPCR results for the DEG subset mirrored those of our TRAP-Seq. Altogether, these data indicate that ABX primarily reduced the astrocytic expression of genes involved in the neuroinflammatory response.

We next performed TRAP-Seq to assess the effects of propionate treatment on APPPS1-21 mice. Here, we found a separation of VHL- and propionate-treated mice in the PCA, with 37% of the variance being explained by the first PC and 16% of the variance being explained by the second PC (Figure 5F). Of note, we observed 2 potential outliers in the PCA plot in the propionate group, which could be a limitation of the downstream analysis. We identified a total of 1,847 DEGs in astrocytes when comparing propionate-treated mice with VHL-treated controls (Figure 5, G and H). Of the 1,847 DEGs, 862 were downregulated in ABX-treated mice and 984 were upregulated (Figure 5, G and H, and Supplemental Table 3). We found via pathway analysis that propionate treatment increased astrocyte neurotrophic pathways, such as synaptic signaling, cognition, learning or memory, social behavior, and response to metal ions (Figure 5I). Furthermore, propionate decreased the expression of genes involved in neurologic disease and inflammatory pathways, including amyotrophic lateral sclerosis, Parkinson disease, fatty acid oxidation, NF-κB signaling, T cell factor–dependent signaling in response to WNT, class I MHC antigen processing and presentation, and the adaptive immune system (Figure 5I).

Since we observed a strong induction of homeostatic astrocyte pathways in ABX- and propionate-treated mouse astrocytes, we next assessed which DEGs upon ABX treatment occurred in the context of propionate treatment in our TRAP-Seq experiments. We found that of 89 DEGs upon ABX treatment, 22 of those genes (25%) were substantially regulated in the same direction in propionate-treated mice (Supplemental Figure 8, A and B). When considering only the direction of fold change, comparing ABX treatment DEGs with propionate treatment DEGs, 75 (84%) of the genes changed in the same direction (Supplemental Figure 8B). Pathway analysis of the 22 genes that were significantly regulated in the same direction in ABX- and propionate-treated mice primarily revealed a reduction in T cell activation and differentiation pathways in ABX- and propionate-treated mouse astrocytes compared with controls (Supplemental Figure 8C). These results suggest that ABX and propionate may reduce astrocyte-mediated T cell activation and differentiation the setting of Aβ amyloidosis.

### ABX and propionate reduce peripheral Th17 cells and IL-17 levels.

**Figure 6:**
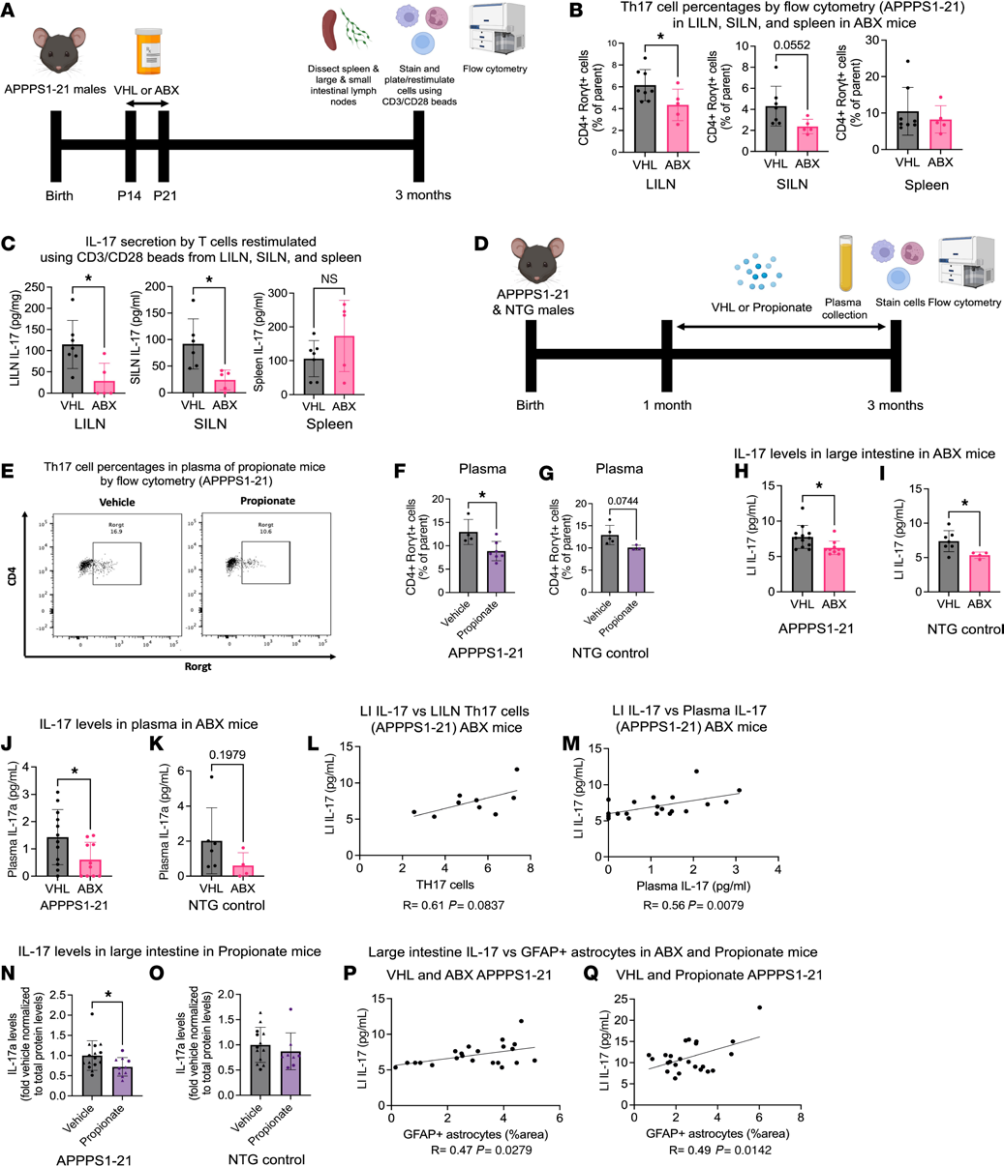
ABX and propionate treatments reduce peripheral Th17 cells and IL-17 levels, which correlate positively with GFAP^+^ reactive astrocytosis in APPPS1-21 mice. (**A**) Schematic depicting the experimental paradigm for ABX-treated APPPS1-21 male mice. (**B**) Th17 cell percentages by flow cytometry in LILN, SILN, and spleen in VHL- and ABX-treated APPPS1-21 mice. (**C**) IL-17 levels in the media of CD3/CD28 bead–restimulated T cells derived from the LILN, SILN, and spleen from VHL- and ABX-treated APPPS1-21 mice. (**D**) Schematic depicting the experimental paradigm for propionate-treated APPPS1-21 and NTG male mice. (**E**) Representative flow cytometry plot depicting a reduction in Th17 cells in the plasma of propionate-treated APPPS1-21 mice. Quantification of Th17 cell percentages by flow cytometry in the plasma of VHL- and propionate-treated (**F**) APPPS1-21 and (**G**) NTG mice. Quantification of IL-17 levels via ELISA in the large intestine of VHL- and ABX-treated (**H**) APPPS1-21 and (**I**) NTG mice. Quantification of IL-17 levels was done via ELISA in the plasma of VHL- and ABX-treated (**J**) APPPS1-21 and (**K**) NTG mice. (**L**) Pearson’s correlation analysis between LILN Th17 cells and LI IL-17 levels in VHL- and ABX-treated APPPS1-21 mice. (**M**) Pearson’s correlation analysis between plasma IL-17 and LI IL-17 levels in VHL- and ABX-treated APPPS1-21 mice. Quantification of IL-17 levels was done via ELISA in the large intestine of VHL- and propionate-treated (**N**) APPPS1-21 and (**O**) NTG mice. Pearson’s correlation analysis between GFAP^+^ astrocyte percentage area and LI IL-17 levels in (**P**) VHL- and ABX-treated APPPS1-21 mice and (**Q**) VHL- and propionate-treated APPPS1-21 mice. Data are expressed as the mean ± SD. *n* = 5–14/group. *P ≤ 0.05, by 2-tailed, unpaired Student’s *t* test. Males are denoted by triangles and females by circles.

It is likely that ABX and propionate reduce reactive astrocytosis and amyloidosis mainly through their effect on peripheral immunity, since neither reaches appreciable concentrations in the brain, and brain cells likely do not express the SCFA receptors according to previous studies (15, 16). Therefore, we first performed flow cytometry on cells stained for cell-surface markers of various immune cell populations from the spleen, large intestinal lymph nodes (LILNs), and small intestinal LNs (SILNs) in ABX- and VHL-treated mice (Figure 6A and Supplemental Figures 9 and 10). We also plated equal amounts of cells from these compartments and restimulated the T cells using CD3/CD28 beads to quantify cytokine release in the media (Figure 6A). Flow cytometry revealed a significant decrease in CD4^+^Rorγt^+^ (Th17) cells in the LILNs, a trend toward reduction in the SILNs, and no change in the spleen in ABX-treated mice compared with control (Figure 6B). Because we found a reduction in Th17 cells, we quantified the levels of their major effector cytokine IL-17 released in the media of CD3/CD28 bead–restimulated cells (Figure 6C). We found that IL-17 levels were significantly decreased in cells restimulated from the LILNs and the SILNs but were not changed in the spleen in ABX-treated mice compared with controls (Figure 6C).

Since we observed that ABX likely have their effect on astrocytes through propionate, we also performed flow cytometry on peripheral blood immune cells in propionate-treated APPPS1-21 and nontransgenic (NTG) mice and controls (Figure 6D and Supplemental Figure 11). Similar to ABX treatment, we found a reduction in Th17 cells in the blood plasma of both APPPS1-21 and NTG mice treated with propionate compared with controls (Figure 6, E–G). Because we found in ABX-treated mice that restimulated T cells produced lower levels of IL-17 in LILNs, we quantified the levels of IL-17 in the large intestinal (LI) lysates and found significantly lower levels of IL-17 in the LI of ABX-treated APPPS1-21 and NTG mice (trend) compared with controls (Figure 6, H and I). Additionally, we observed lower levels of IL-17 in plasma isolated from ABX-treated APPPS1-21 (Figure 6J) and NTG (trend) mice compared with controls (Figure 6K). Interestingly, LI IL-17 levels trended toward a positive correlation with LILN TH17 levels (*P* = 0.0837) (Figure 6L), suggesting that IL-17 was probably coming from Th17 cells rather than another source. LI IL-17 levels also correlated positively with plasma IL-17 levels (Figure 6M), indicating that LI-derived IL-17 may also get into the plasma and influence circulating immune or CNS cells. Since we found lower levels of IL-17 in the LI of ABX-treated mice, we predicted we would find a similar reduction in propionate-treated mice. Indeed, we found a reduction of LI IL-17 in propionate-treated APPPS1-21 but no change in NTG mice compared with controls (Figure 6, N and O). Since we hypothesized that ABX- and propionate-mediated reductions in Th17 and IL-17 levels may be the mechanism through which GFAP^+^ reactive astrocytosis is reduced in both models, we assessed correlations between LI IL-17 levels and GFAP^+^ astrocytosis and found significant positive correlations in both treatments (Figure 6, P and Q). Additionally, we asked whether there were any changes in immune cell infiltration into the brain parenchyma following propionate treatment. We treated male and female APPPS1-21 mice from 3 months of age to 6 months of age with propionate and assessed immune cell populations in the brain using flow cytometry. We chose to treat the mice from 3–6 months because a propionate pilot experiment showed no Th17 cell infiltration at 3 months of age. We found no changes in Th17 cells or other cell populations following propionate treatment from 3–6 months (Supplemental Figure 12), suggesting that propionate does not influence Th17 cell infiltration into the brain during amyloidosis. We postulate that propionate-induced decreases in peripheral IL-17 traveling to the brain are responsible for reduced cerebral reactive astrocytosis and amyloidosis.

Overall, these results suggest that ABX- and propionate-mediated reductions in peripheral Th17 cells and IL-17 levels may lead to decreases in GFAP^+^ reactive astrocytosis and amyloidosis. To determine the relevance of Th17 biology to human AD, we assessed the expression of gene markers (17) of Th17 cells in a recent PBMC scRNA-Seq dataset by Ramakrishnan and colleagues (17, 18). Interestingly, markers of Th17 cells, such as *RORC*, *KLRB1*, *CCR6*, were increased in patients with AD compared with healthy controls (Supplemental Figure 13, A–C and F). Gaublomme and colleagues previously used scRNA-Seq to identify G protein–coupled receptor 65 and Fc µ receptor as regulators of Th17 pathogenicity (19). Expression of these markers was also significantly increased in patients with AD compared with healthy controls (Supplemental Figure 13, D–F). Together these data suggest there may be increased circulating pathogenic Th17 cells in AD that contribute to disease progression.

### Propionate-mediated reduction in GFAP^+^ reactive astrocytosis and amyloidosis is dependent on IL-17.

**Figure 7:**
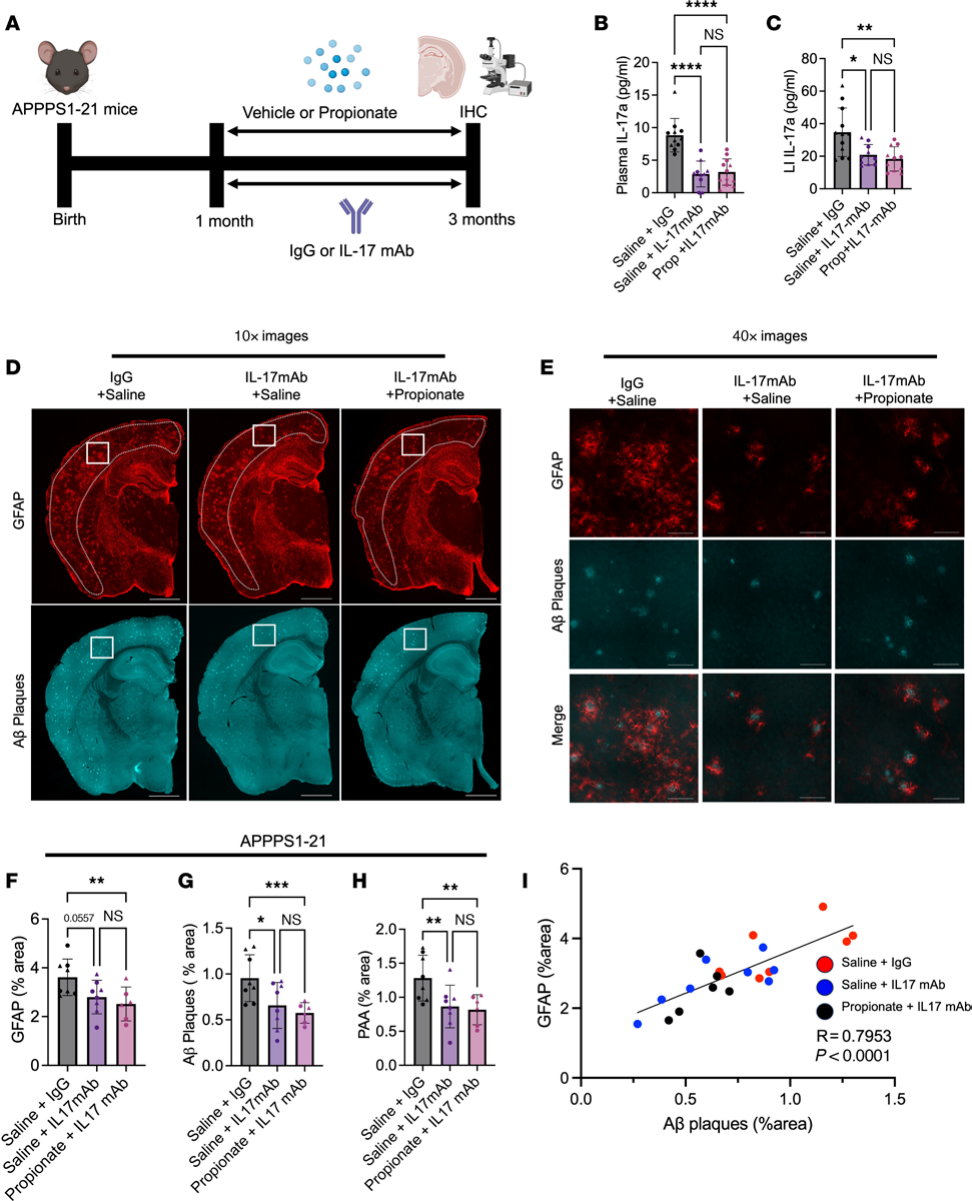
Propionate-induced reductions in GFAP^+^ reactive astrocytosis and Aβ amyloidosis are dependent on IL-17 signaling in APPPS1-21 mice. (**A**) Schematic depicting the experimental paradigm. (**B**) Plasma IL-17 levels in APPPS1-21 male and female mice treated with saline plus IgG, saline plus IL-17 mAb, or propionate plus IL-17 mAb. (**C**) LI levels of IL-17 in saline plus IgG, saline plus IL-17 mAb, and propionate plus IL-17 mAb groups. (**D** and **E**) Representative images of GFAP^+^ astrocytes and Aβ plaques in saline plus IgG, saline plus IL-17 mAb, and propionate plus IL-17 mAB groups. Scale bars: 1,000 μm (original magnification, ×10; **D**) and 100 μm (original magnification, ×40; **E**). Quantification of the percentage of areas of (**F**) GFAP^+^ astrocytes, (**G**) Aβ plaques, and (**H**) PAAs in the saline plus IgG, saline plus IL-17 mAb, and propionate plus IL-17 mAb groups. (**I**) Pearson’s correlation analysis between the area percentages of GFAP^+^ astrocytes and Aβ plaques. Data are expressed as the mean ± SD. *n* = 6–12/group. Four sections were used per animal. *P ≤ 0.05, **P ≤ 0.01, ***P ≤ 0.001, and ****P ≤ 0.0001, by 2-way ANOVA. Males are denoted by triangles and females by circles. Dotted lines indicate the area of cortex analyzed.

To determine whether the propionate-mediated reductions in reactive astrocytosis and amyloidosis were dependent on IL-17 signaling, we treated male and female APPPS1-21 mice with an IL-17 mAb or IgG1 control to reduce the levels of peripheral IL-17. The IL-17 mAb-administered mice were simultaneously treated with saline or sodium propionate (Figure 7A). Compared with mice treated with saline plus IgG control, mice treated with saline plus IL-17 mAb demonstrated 69% and 40% reductions of IL-17 in plasma and LI, respectively (Figure 7, B and C). Similarly, mice treated with propionate plus IL-17 mAb had 64% and 47% decreases in IL-17 in the plasma and LI, respectively, compared with saline plus IgG control (Figure 7, B and C). Interestingly, propionate plus IL-17 mAb treatment did not reduce IL-17 levels in the plasma or LI more than saline plus IL-17 mAb control, which we speculate is because of the already significant reduction of IL-17 due to IL-17 mAb treatment at the maximum lowering amount achievable by propionate. Importantly, both the saline plus IL-17 mAb and propionate plus IL-17 mAb groups had a lower Aβ plaque burden, GFAP^+^ reactive astrocytosis, and PAAs compared with the saline plus IgG control group, suggesting that IL-17 reduction alone reduced both GFAP^+^ reactive astrocytosis and Aβ amyloidosis (trend in the saline plus IL-17 mAb group for GFAP; Figure 7, D–H). More important, we observed no change in Aβ amyloidosis, GFAP^+^ reactive astrocytosis, or PAAs when comparing the saline plus IL-17 mAb and propionate plus IL-17 mAb groups (Figure 7, D–H). This result suggests that the propionate-induced reduction in Aβ amyloidosis and GFAP^+^ reactive astrocytosis was dependent on IL-17 signaling. Furthermore, Pearson’s correlation analysis demonstrated a positive correlation between GFAP^+^ astrocytosis and Aβ plaque burden, suggesting that Aβ plaque burden may be influenced by reactive astrocytosis, at least in part (Figure 7I).

## Discussion

GMB composition has been shown to be altered in human AD, indicating that it may play a role in AD pathogenesis and progression (2). Several studies have shown that ABX-induced GMB alteration reduces Aβ plaques and microglial activation in AD model mice (3–6, 20). While microglia are important for responding to Aβ pathology in AD, astrocytes also play an important role. We previously showed that ABX-mediated GMB perturbation reduces reactive astrocytosis and induces homeostatic morphologic changes through microglial-independent and -dependent mechanisms, respectively, in the APPPS1-21 model of amyloidosis (9).

In the current study, we investigated whether ABX-mediated changes in reactive astrocytosis and amyloidosis occur through changes in microbial metabolites. Using GC-MS, we found that the SCFA propionate was selectively increased in the plasma of male APPPS1-21 mice after ABX treatment compared with controls. Propionate has been found to be reduced in mouse models of AD and patients with AD (21–23). We previously found that the bacterial genus *Akkermansia*, which produces propionate at high levels, was also selectively increased in male APPPS1-21 mice compared with controls, suggesting that ABX-mediated increases in *Akkermansia* may be responsible for elevated levels of plasma propionate (9).

We found that propionate treatment phenocopied ABX treatment, in that it reduced GFAP^+^ astrocytosis and amyloidosis. Interestingly, there did not appear to be sex differences following propionate treatment, as has been observed after ABX treatment. This may be due to the lack of propionate production following ABX treatment in females. This could be caused by the failure of ABX to induce *Akkermansia* or other propionate-producing bacteria and not in the downstream response(s) to propionate.

Using snRNA-Seq on mouse cortices from APPPS1-21 mice that were treated with ABX, we found reductions in neuroinflammatory and neurodevelopmental astrocyte pathways and subclusters and an increase in neurotrophic astrocyte pathways and subclusters. Similarly, TRAP-Seq revealed reductions in neuroinflammatory and CNS disease–associated astrocyte pathways and an increase in neurotrophic astrocyte pathways in ABX- and propionate-treated APPPS1-21 mice compared with control mice.

According to previous studies, ABX and propionate do not reach high concentrations in the brain parenchyma. Also, we found nearly undetectable levels of propionate in the brains of mice treated with exogenous propionate. Therefore, it is more likely that propionate influences peripheral immunity, which in turn affects reactive astrocytosis (16, 24), rather than a direct effect of propionate on the brain. We found reductions in peripheral Th17 cells and IL-17 levels in APPPS1-21 mice that were treated with ABX or propionate. It is well known that Th17 cells are regulated in part by the GMB (25). Propionate has previously been shown to reduce naive T cell differentiation into Th17 cells in a GPR43 receptor–dependent manner (26, 27). Therefore, we contend that ABX-mediated increases in propionate reduce the differentiation of peripheral Th17 cells and subsequent IL-17 release.

Furthermore, we found that propionate did not directly alter Th17 infiltration into the brain, suggesting that the major direct effect was on peripheral, rather than central, immunity. It is still an open question as to whether ABX treatment or propionate alters Th17 cell infiltration into the brain in later stages of disease, which will need to be addressed in future studies.

Interestingly, the levels of IL-17 in the LI correlated positively with GFAP^+^ astrocytosis in both ABX- and propionate-treated mice. Importantly, using an IL-17 mAb, we found that propionate-induced reductions in GFAP^+^ astrocytosis and Aβ amyloidosis were dependent on IL-17. These results suggest that propionate controls peripheral IL-17 signaling to regulate reactive astrocytosis and affect amyloidosis.

We believe that ABX- and propionate-induced restoration of an astrocyte homeostatic state occurs through a reduction in peripheral IL-17 signaling. It is possible that this leads to an increased ability of astrocytes to directly phagocytose Aβ plaques and instruct microglia to increase their phagocytosis of Aβ plaques. Relatedly, Cao and colleagues found that IL-17 reduced the phagocytic ability of BV-2 microglia (28). It is likely that a similar phenomenon occurs with astrocytes, as it is well known that reactive astrocytes have a reduced phagocytic ability (10). Furthermore, we previously found that complement C3 expression in astrocytes was decreased by ABX treatment, and C3 derived from astrocytes has been shown to bind to the C3a receptor on microglia and impede microglial phagocytosis of Aβ (9, 29).

There are several limitations of our study. One limitation is that mice were treated with ABX from P14-21, which simulates early-life GMB perturbation rather than later-life changes. This was done because the microbiome is more dynamic and amenable to large-scale changes in early life (5, 6, 30). It will be important for future studies to elucidate the role of late-life GMB perturbation in AD-relevant phenotypes. Here, we identify propionate as a likely mediator of ABX-induced reductions in reactive astrocytosis and amyloidosis. However, it is possible that there may be other gut-derived byproducts or metabolites that mediate the ABX phenotype that were not tested in the current study.

Additionally, although we found that ABX treatment increased propionate, it should be noted that Seo and colleagues found a decrease in cecal propionate and other SCFAs following ABX treatment in Tau PS19 mice with differing APOE genotypes at 40 weeks of age (7). We speculate that the differences between the work by Seo et al. and our study are likely due to combined effects of the use of amyloid versus tau mouse models, plasma versus cecal sampling, and 9-week versus 40-week ages at which SCFAs were measured, and differences in bacteria after ABX treatment.

Another limitation of our study is that the snRNA-Seq was performed on mice housed at the University of Chicago rather than Northwestern University. We have found that the mice housed at the different sites appeared to have altered fecal microbiome profiles. However, it has been previously shown that, although GMBs between facilities may be different, similar effects on amyloidosis and neuroinflammation are achieved when using the same combination of ABX or other GMB-targeted interventions (3–7, 9, 13).

Although our study has found that propionate reduces reactive astrocytosis and amyloidosis, some other studies have found that combinatorial SCFAs, including acetate, propionate, and butyrate, increase amyloidosis and neuroinflammation in germ-free mice (31). However, it has been shown that acetate by itself increases both amyloidosis and neuroinflammation (24). Comparison of each of the SCFAs administered by themselves on these phenotypes has never been reported, to our knowledge. It is likely that SCFAs have different effects and that the effects may be dose and timing dependent in amyloid models of AD. We suspect that the SCFA combinatorial studies dilute the protective effects of propionate due to the inclusion of high levels of acetate and excess total SCFA levels. Additionally, germ-free mice have several developmental defects, which makes their study in isolation come with several inherent caveats (15, 32).

**Figure 8:**
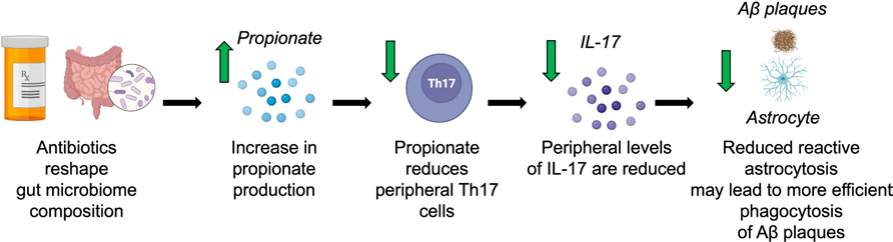
Hypothesis of ABX-mediated GBM control of reactive astrocytosis and amyloidosis. ABX reshape gut microbial composition (i.e., increased *Akkermansia*), which leads to changes in the levels of gut-derived metabolites, such as an increase in propionate. Propionate reduces peripheral Th17 cells and IL-17 production in the periphery, which likely leads to lower concentrations of IL-17 in the CNS. Since IL-17 activates astrocytes, which may compromise their ability to phagocytose Aβ plaques, lower IL-17 levels reduce reactive astrocytes and decrease Aβ plaques. The propionate-induced reduction is dependent on IL-17 signaling.

Overall, we found that ABX-mediated GMB perturbation increased propionate levels in the context of amyloidosis (Figure 8). Both ABX and propionate administration reduce the levels of peripheral Th17 cells, IL-17, and GFAP^+^ reactive astrocytosis in the brain (Figure 8). Multiomics sequencing revealed that both ABX and propionate treatment led to a reduction in neuroinflammation and an increase in neurotrophic astrocyte transcriptional programs. These astrocyte changes corresponded with reductions in amyloidosis. We postulate that a GMB-induced restoration of astrocyte homeostasis restrains Aβ plaques through increased phagocytosis (Figure 8). This reduction in Aβ plaques as well as an increase in neurotrophic support from astrocytes may result in improved neuronal and synaptic health. Our study clarifies the molecular mechanisms of GMB and peripheral immunity contributions to reactive astrocytosis and AD. It is possible that the mechanisms elucidated in this study could be harnessed for the development of therapeutic strategies for AD treatment. Exogenous sodium propionate treatment given to patients with multiple sclerosis over 3 years reduced the annual relapse rate, disability stabilization, and brain atrophy compared with controls (33). Our findings suggest that sodium propionate administration may also be efficacious for patients with AD. Additionally, increasing endogenous propionate production via *Akkermansia* administration, a high-fiber diet, or propionate producing engineered probiotics may prevent the development of amyloidosis and reactive astrocytosis.

## Methods

### Sex as a biological variable.

It has been repeatedly shown that ABX treatment only has effects on amyloidosis and neuroinflammation in male but not female amyloid model mice (5, 6, 9). For this reason, we only used male mice for ABX treatment, except in our GC-MS experiment. For our propionate treatment groups, we used both male and female mice because, while ABX treatment did not influence amyloidosis or neuroinflammation in females, it was conceivable that propionate treatment may have effects on both sexes.

### Animal housing and handling.

APPPS1-21 and *Aldh1l1*-EGFP *Rpl10a* bacTRAP mice were housed in the Northwestern University Center for Comparative Medicine in a specific pathogen–free environment. To generate APPPS1-21^+^ *Aldh1l1*-EGFP *Rpl10a*^+^ mice, hemizygous male APPPS1-21 breeders were crossed with homozygous female *Aldh1l1*-EGFP *Rpl10a* breeders. For snRNA-Seq, APPPS1-21 mice were housed in the animal research center at the University of Chicago (the only experiment for which mice housed at the University of Chicago were used).

### ABX treatment.

APPPS1-21 male and female mice and WT controls were orally gavaged with 200 μL ABX cocktail (4 mg/mL kanamycin, 0.35 mg/mL gentamicin, 8,500 U/mL colistin, 2.15mg/mL metronidazole, 0.45 mg/mL vancomycin in autoclaved water) or water VHL from P14 to P21 as previously described (5, 6, 9). Mice were randomly assigned to ABX or the VHL water group. Mouse cages were changed each day from P14 to P21 to avoid consumption of feces from the previous day. Mice were sacrificed and perfused at 9 weeks or 3 months of age, as APPPS1-21 mice at these ages have sufficient neuroinflammation and amyloidosis in the brain cortex, as demonstrated in previous similar studies (5, 6, 9).

### Propionate administration.

APPPS1-21 male and female mice and WT controls were given drinking water containing 150 mM sodium propionate (MilliporeSigma, P1880) or 150 mM NaCl (Thermo Fisher Scientific, S271-1) from 1 month of age to 3 months of age. Doses were selected on the basis of previous studies (24, 34). Mice were randomly assigned to the propionate or NaCl group. Mice were perfused and sacrificed at 3 months of age.

### IL-17 mAb administration.

APPPS1-21 male and female mice were randomly assigned and treated with 150 mM saline (Thermo Fisher Scientific, S271-1) or 150 mM sodium propionate (MilliporeSigma, P1880) in their water bottles from 1 month to 3 months of age. In addition, both saline- and propionate-treated mice received anti–mouse IL-17A mAb (BE0173, Bio X Cell) every third day at a dose of 10 mg/kg from 1 month to 3 months of age, similar to previous studies (35–37). A randomly assigned subset of saline-treated control mice were given mouse IgG1 isotype control antibody (BE0083, Bio X Cell) following the same dosing schedule. IL-17A levels in plasma and LI were measured using a high-sensitivity IL-17 ELISA (MHS170, R&D Systems). Mice were randomly assigned to the treatment groups.

### Perfusion and tissue preservation.

Mice were transcardially perfused at 3 months of age with perfusion buffer containing 20 mg/mL phenylmethylsulfonyl fluoride, 5 mg/mL leupeptin, 200 nM sodium orthovanadate, and 1 M dithiothreitol in 1× PBS. Following perfusion, the left hemisphere of the brain was collected for IHC analysis, and the right hemisphere was subdissected into cortex, hippocampus, midbrain, and cerebellum and flash-frozen in liquid nitrogen. For TRAP-Seq, prior to freezing tissues, all brain regions were washed with a dissection buffer as described by Heiman et al. (14) (1× HBSS, 2.5 mM HEPES-KOH, 35 mM glucose, 4 mM NaHCO3, 100 μg/mL cycloheximide in RNase-free water). Dorsal cortex was cut into an approximately 70 mg for TRAP-Seq and the rest of the ventral cortex was used to make protein lysates. Left brain hemispheres were postfixed in 10% formalin and cryopreserved in 30% sucrose. Left hemispheres were cut into 40 μm sections to be used to for IHC. LIs were also dissected and cleared with PBS and flash-frozen in liquid nitrogen.

### IHC.

Four comparable sections containing cortex were used for each IHC experiment. Sections were first washed 3 times in 1× TBS buffer for 5 minutes with agitation. The sections were then incubated in 16 mM glycine with 0.25% triton TBS for 1 hour at room temperature (RT). After a set of three 5-minute washes in TBS, sections were incubated in donkey anti–mouse IgG to minimize background. After three 5-minute washes in TBS, sections were incubated overnight in primary antibodies diluted in 1% BSA in 0.25% triton TBS (1% BSA buffer). The following day, sections were washed 3 times for 10 minutes in 1% BSA buffer. The sections were then incubated in secondary antibodies in 1% BSA buffer for 1 hour at RT in the dark. After three 5-minute TBS washes, sections were mounted in TBS on Diamond White Glass Charged Slides (Globe Scientific, 1358T). Coverslips (Thermo Fisher Scientific, 3421) and were applied to slides with ProLong Gold reagent (Invitrogen, Thermo Fisher Scientific, P36930). Section selection was performed by a person blinded to treatment groups and genotypes.

### Microscopy and quantification.

Wide-field microscopy images were taken in the Northwestern University Center for Advanced Microscopy and Nikon Imaging Center on a Nikon Ti2 widefield microscope with a ×10 or ×40 air objective. Multiple images were automatically taken and stitched together by the NIS elements software to construct ×10 images of each whole brain section. The images were saved as ND2 files and quantified using the Nikon NIS elements general analysis tool. Regions of interest of the whole cortex in each section were traced using the NIS region of the interest polygon tool. Thresholds for a positive signal for each stain were defined in the NIS elements software, and then signal was automatically quantified using the batch tool. Image acquisition, image tracing, and quantifications were performed by a person blinded to treatment groups and genotypes.

### Antibodies for IHC and immunoblotting.

For IHC, the following antibodies were used: chicken anti-GFAP (1:1,000, Abcam, Ab4674) and mouse anti-Aβ 3D6 (1:1,000, Elan Pharmaceuticals). For immunoblotting, the following antibodies were used: rabbit anti-GFAP (1:5,000, MilliporeSigma, G9269); mouse anti-Synaptophysin (1:1,000, Abcam, ab8049); mouse anti-PSD95 (1:2,000, Abcam, ab192757)l and rabbit anti-GAPDH (1:5,000, Cell Signaling Technology, 2118).

### GC-MS procedure and data analysis.

SCFAs were derivatized as described by Haak et al. (38), with the following modifications. The metabolite extract (100 μL) was added to 100 μL of 100 mM borate buffer (pH 10) (Thermo Fisher Scientific, 28341); 400 μL of 100 mM pentafluorobenzyl bromide (MilliporeSigma; 90257) in acetonitrile (Thermo Fisher Scientific, A955-4); and 400 μL *n*-hexane (Acros Organics, 160780010) in a capped mass spectrometric autosampler vial (Microliter, 09-1200). Samples were heated in a thermomixer C (Eppendorf) to 65°C for 1 hour while shaking at 1,300 rpm. After cooling to RT, samples were centrifuged at 4°C, 2,000*g* for 5 minutes, allowing for phase separation. The hexanes phase (100 μL) (top layer) was transferred to an autosampler vial containing a glass insert and the vial was sealed. Another 100 μL of the hexanes phase was diluted with 900 μL *n*-hexane in an autosampler vial. Concentrated and diluted samples were analyzed using GC-MS (Agilent Technologies, 7890A GC system, Agilent 5975C MS detector) operating in negative chemical ionization mode, using a HP-5MSUI column (30 m × 0.25 mm, 0.25 μm; Agilent Technologies, 19091S-433UI), methane as the reagent gas (99.999% pure), and 1 μL split injection (1:10 split ratio). The oven ramp parameters were as follows: a 1-minute hold at 60°C and 25°C per minute up to 300°C with a 2.5-minute hold at 300°C. The inlet temperature was 280°C, and the transfer line was 310°C. Data analysis was performed using MassHunter Quantitative Analysis software (version B.10, Agilent Technologies) and confirmed by comparison with authentic standards. Normalized peak areas were calculated by dividing the raw peak areas of the targeted analytes by the averaged raw peak areas of the internal standards.

### Single-nucleus isolation, sequencing, and analysis.

Single-nucleus suspensions were generated from the cortices of vehicle- and ABX-treated APPPS1-21 male mice housed at the University of Chicago using the 10X Genomics Chromium Nuclei Isolation Kit (PN-1000494). After isolation, nuclei were loaded into the 10X Genomics Chromium Controller with a target of 10,000 nuclei per sample. Gel bead-in emulsions (GEM) generation and library preparation were performed according to the 10X Chromium Next GEM Single Cell 3′ Reagent Kits version 3.1 chemistry workflow. Libraries were pooled and sequenced on an Illumina NovaSeq 6000, and reads were demultiplexed and aligned to the genome using CellRanger. A total of 109,581 nuclei were sequenced at an average post-normalization read depth of 31,674 reads per cell and 1,736 genes per cell. Data then underwent quality control using SoupX and DoubletFinder R packages. Quality controlled data were then integrated, and multidimensional reduction using uniform manifold approximation and projection (UMAP) plots were produced using Seurat. Differential expression analysis between clusters was performed using Seurat’s FindMarkers function. UMAP was manually annotated using the highly expressed genes in each cluster. Analysis of DEGs between the VHL- and ABX-treated groups was performed using Seurat’s FindMarkers function using the masked augmentation subspace training (MAST) algorithm from the R package MAST. The Benjami-Hochberg (BH) method was used to correct for multiple testing. An FDR corrected *P* value of 0.01 with log_2_ fold change of 0.25 was the cutoff for significance. Astrocyte clusters were subclustered using the subset function in Seurat. Astrocytes were subclustered using the first 30 PCs at a resolution of 0.1. DEG analysis between astrocyte subclusters was performed using Seurat’s FindMarkers function using the MAST algorithm from the R package MAST (full results can be found in Supplemental Table 1). The BH method was used to correct for multiple testing in the astrocyte subcluster DEG analysis. An FDR-corrected *P* value of 0.001 with a log_2_ fold change of 0.25 was the cutoff for significance for pathway analysis, which was performed using Metascape.

### TRAP.

*Aldh1l1*-EGFP/*Rpl10a* bacTRAP mice incorporate an EGFP-RPL10a ribosomal fusion protein targeted to the astrocyte-specific *Aldh1l1* gene. This animal model enables cell labeling, sorting, and affinity purification of astrocyte-specific polysomal RNAs (14). To capture EGFP^+^ polysomal complexes in astrocytes for TRAP-Seq, an anti-GFP affinity matrix (AM) was prepared prior to homogenization of cortical tissue using a previously published protocol (14). Streptavidin MyOne T1 Dynabeads were resuspended, and 450 μL/purification was added to the AM tube. Beads were collected by a magnet and washed with 1× PBS. A 180 μL/purification of biotinylated protein L was then added to the beads, and the mixture was incubated on a tube rotator for 35 minutes. The protein l–coated beads were captured on a magnet and washed 5 times with 1× PBS containing 3% (w/v) IgG and protease-free BSA. Anti-GFP antibodies (75 μg) (Htz-GFP-19F7 and Htz-GFP-19C819F7 obtained from Memorial Sloan Kettering Cancer Center Antibody and Bioresource Core Facility) in low-salt buffer (20 mM HEPES KOH, 150 mM KCl, 10 mM MgCl2, and 1% [vol/vol] NP-40 in RNase-free water) was then added to the bead mixture and incubated on a tube rotator for 1 hour. After antibody binding, the final AM was washed 3 times with low-salt buffer. After washing, the AM was resuspended in a volume of low-salt buffer, such that each IP received an aliquot of 200 μL. Following AM preparation, flash-frozen mouse cortices (washed in dissection buffer before freezing) were homogenized in 1.5 mL tissue lysis buffer (20 mM HEPES KOH, 150 mM KCl, 10 mM MgCl2, EDTA-free protease inhibitor tablet, 0.5 mM DTT, 100 μg/mL cycloheximide in RNase-free water) in a glass tube with an electric homogenizer (Glas-Col, 099CK5424) at 900 rpm with 15 full strokes. Lysates were transferred into prechilled microcentrifuge tubes on ice. Post-nuclear supernatants were prepared (S2) by centrifugation at 4°C for 10 minutes at 2,000*g*. S2 was transferred to a new, prechilled microcentrifuge tube on ice. A 1/9 sample volume of 10% NP-40 to S2 (final concentration 1%) was added and mixed gently by inverting the tube. A 1/9 sample volume of 300 mM DHPC was added to S2 (final concentration 30 mM) and mixed gently by inverting the tube, and the mixture was incubated on ice for 5 minutes. Post-mitochondrial supernatants (S20) were prepared by centrifugation at 4°C for 10 minutes at 20,000*g*. S20 was transferred to a new, prechilled microcentrifuge tube. Freshly resuspended AM (200 μL) was added to each S20 sample. Bead complexes were washed 4 times with high-salt buffer (20 mM HERPES, 350 mM KCl, 10 mM MgCl, 1% NP-40 in RNase-free water). RNA was isolated from beads using 100 μL NanoPrep lysis buffer (from Stratagene Absolutely RNA Nanoprep kit). RNA cleanup was performed according to the kit’s instructions.

### TRAP-Seq and analysis.

The quality of RNA isolated from the TRAP purifications was assessed using an Agilent Bioanalyzer. All RNA used for downstream sequencing had a RNA integrity number score above 7. mRNA libraries were generated from TRAP-purified RNA using the Illumina Stranded mRNA preparation kit. Prepared libraries were sequenced on an Illumina HighSeq 4000. The quality of reads, in FASTQ format, was evaluated using FastQC. Reads were trimmed to remove Illumina adapters from the 3′ ends using cutadapt (39). Trimmed reads were aligned to the *Mus musculus* genome (mm39) using STAR (https://github.com/alexdobin/STAR). Read counts for each gene were calculated using htseq-count (40) in conjunction with a gene annotation file for mm39 obtained from Ensembl (http://useast.ensembl.org/index.html). Normalization and differential expression were calculated using DESeq2, which uses the Wald test (https://bioconductor.org/packages/release/bioc/html/DESeq2.html). The cutoff for determining significant DEGs was an FDR-adjusted *P* value of less than 0.1 using the BH method. Pathway analysis was performed using Metascape. (Full results can be found in Supplemental Tables 2 and 3 for ABX and propionate, respectively.) Raw sequencing files are available in the NCBI Gene Expression Omnibus (GEO) database (GEO GSE295458).

### qPCR analysis.

RNA isolated via the TRAP protocol as described above was converted to cDNA using Invitrogen Superscript IV VILO Mastermix Kit (Thermo Fisher Scientific, catalog 11766050). The master mixes for qPCR were made using TaqMan Fast Advanced Master Mix (Thermo Fisher Scientific, catalog 4444556) and TaqMan single-tube gene expression assay primers against *Gfap* (Thermo Fisher Scientific, Mm01253033_m1), *Serpina3n* (Thermo Fisher Scientific, Mm00776439_m1), *Irgm1* (Thermo Fisher Scientific, Mm00492596_m1), *Bag3* (Thermo Fisher Scientific, Mm00443474_m1), *Fbxo32* (Mm00499523_m1), *Cyp7b1* (Thermo Fisher Scientific, Mm00484157_m1), and *Fezf2* (Thermo Fisher Scientific, Mm01320619_m1). Experimental primers were normalized to *Gapdh* (Thermo Fisher Scientific, Hs02786624). Reactions were run on a 96-well plate in an Applied Biosystems QuantStudio 7 flex machine (catalog 448701) at the Northwestern NUSeq core facility. The protocol used for amplification contained a 2-minute hold step at 50°C, a 2-minute hold step at 95°C, and 40 cycles of 1 second at 95°C followed by 20 seconds at 60°C. Genes selected for this qPCR validation analysis were DEGs shared between ABX and propionate analysis, for which validated primers were available through Thermo Fisher Scientific (≥2 citations).

### Flow cytometry.

Mesenteric lymph nodes (LILN and SILN-draining), spleen, or plasma were isolated from mice. LNs were digested for 45 minutes at 37°C in DMEM with collagenase IV (1 mg/mL; Worthington). LNs and spleens were homogenized using syringe plungers on 70 mM strainers. Spleens were lysed using 3 mL ACK lysis buffer (Gibco, Thermo Fisher Scientific) for 5 minutes and quenched with 30 mL DMEM. Cells were counted using a cell counter (Contessa). Single-cell suspensions of LNs, spleens, or plasma were plated in U-bottomed, 96-well plates. Cells were washed in PBS and stained for 15 minutes at 4°C with Live/Dead Fixable Dye (Thermo Fisher Scientific) and 1:200 Fc Block (BD Biosciences). Cells were washed in PBS plus 2% FBS and stained for 30 minutes at 4°C with surface antibodies at a 1:200 dilution. For intracellular cytokine staining, cells were fixed in 2% paraformaldehyde and permeabilized in 0.1% saponin. Cells were washed twice in permeabilization buffer and stained in permeabilization buffer for 30 minutes at 4°C. Cells were washed twice in permeabilization buffer and resuspended in PBS plus 2% FBS for analysis. For transcription factor staining, cells were fixed and permeabilized for 50 minutes at 4°C (FoxP3/Transcription Factor Kit, eBioscience). Cells were then washed twice and stained for 1 hour in permeabilization buffer. Cells were washed twice and resuspended in PBS plus 2% FBS. Data were acquired on an LSRFortessa or BD FACSymphony (BD Biosciences) system and analyzed by FlowJo software. For information on markers of cell populations see Supplemental Table 4.

### Ex vivo restimulation.

For 3-day ex vivo restimulation, 320,000 splenocytes or 120,000 LN cells were plated in a 96-well, round-bottomed plate, and cells were stimulated with 2 mL CD3/CD28 beads. After 3 days, the supernatant was taken for cytokine analysis via ELISA.

### Protein lysate generation and ELISAs.

Large intestine and brain lysates were generated by homogenizing LI tissue (washed with PBS during initial dissection) using a hand-held electronic homogenizer in 1 mL RIPA buffer (50 mM tris, 0.15 M NaCl, 1% octylphenoxypolyethoxyethanol [IGEPAL], 0.1% SDS, and 0.5% sodium deoxylate at pH8) containing protease inhibitor (catalog 535140, Calbiochem) and phosphatase inhibitor (catalog 78427, Thermo Fisher Scientific) cocktails. Samples were incubated for 30 minutes on ice and then sonicated for 20 seconds each. The samples were then centrifuged at 14,000*g* for 30 minutes, and the supernatant was collected (RIPA soluble fraction). The protein concentration was determined using the Pierce BCA Protein Assay kit (Thermo Fisher Scientific). IL-17 ELISAs were performed using both regular (catalog BMS6001, Thermo Fisher Scientific) and high-sensitivity kits (catalog MHS170, R&D Systems).

### Mouse brain flow cytometry.

APPPS1-21 mice were treated with 150 mM sodium propionate (MilliporeSigma, P1880) or 150 mM NaCl (Thermo Fisher Scientific, S271-1) from 3 months of age to 6 months of age. Mouse brains were harvested, and the left hemibrain was dissected for brain cell isolations. Specifically, the left hemisphere was injected with 3 mL digestion buffer consisting of 2.5 mg/mL Liberase TL and 1 mg/mL DNase I in HBSS (Roche), morcellated, and rapidly transferred to C-tubes. Tissue in C-tubes was then dissociated using a MACS dissociator according to the manufacturer’s instructions. The dissociated tissue was strained through a 40 μm nylon mesh strainer and washed with 100 mL autoMACs running buffer per brain. The C-tubes, MACS dissociator, and autoMACs running buffer were all purchased from Miltenyi Biotec. Cell debris was removed using Debris Removal Solution (130-109-398, Miltenyi Biotec), and a brain-derived single-cell suspension was made according to the manufacturer’s guidelines.

Cell-surface staining was performed using the following antibodies: Fixable Viability Dye eFluor 506 (65-0866-14, Invitrogen, Thermo Fisher Scientific); CD45 BUV661 (1:400; 612975, clone: 30-F11, BD Biosciences); CD11b-APC-Cy7 (1:1,000; A15390, clone: M1/70; Invitrogen, Thermo Fisher Scientific); Ly6C BV421 (1:1,000; 562727, clone: AL-21, BD Biosciences); TCRgd BUV737 (1:200; 748991, clone: GL3, BD Biosciences);, CD3e BUV396 (1:100, 563565, clone: 145-2C11; BD Biosciences); CD8 PerCP-Cy5.5(1:67; 1299159, clone: 53-6.7, BD Biosciences); CD4 AF700 (1:333; 557956, clone: RM4-5, BD Biosciences); CD64 BV786 (1:83; 741024, clone: X54-5/7.1, BD Biosciences);, MHCII BV605 (1:200; 563413, clone: M5/114.15.2, BD Biosciences); and CD25 PE-Cy7 (1:200; 552880, clone: PC61, BD Biosciences). For intracellular staining, the Foxp3/Transcription Factor Staining Buffer Set (00-5523099; eBioscience) was used following the manufacturer’s guidelines. The following antibodies for intracellular staining were used: Tbet BV711 (1:40; 644819, clone: 4B10, BioLegend); GATA3 PE (1:100; 12-9966-42, clone: TWAJ, Invitrogen, Thermo Fisher Scientific); and RORγt Alexa Fluor 647 (1:400; 562682, clone: Q31-378; BD Biosciences). Stained cells were then analyzed on a BD FACSymphony flow cytometer (BD Biosciences), and analysis was performed using FlowJo software, version 10.0. CD4^+^CD25^+^FOXP3^+^ Tregs, TCRgd^+^ T cells, or CD4^+^ GATA3^+^ Th2 cells were not detected.

### Immunoblotting.

Protein lysates were diluted to 2 μg/μL, and for immunoblots, 18 μg total protein was mixed with 4× Laemmli buffer (catalog 1610747, Bio-Rad) and heated at 95°C for 10 minutes. The samples were then run on 4%–12% Criterion XT Bis-Tris polyacrylamide gels (catalog 3450126, Bio-Rad) in 2-(N-morpholino) ethanesulfonic acid. Gels were transferred onto 0.45 μm nitrocellulose membranes (catalog 1620167, Bio-Rad) using the Bio-Rad Trans-blot Turbo Transfer System. Membranes were briefly incubated in 0.1% Ponceau solution to assess transfer quality. After three 5-minute washes in TBST, membranes were incubated in 5% milk in TBST for 1 hour. Primary antibodies were incubated in 5% milk overnight at 4°C, and secondary antibodies were incubated in 5% milk for 1 hour at RT. After 2 TBST and 1 TBS 5-minute washes, membranes were incubated in SuperSignal West Pico Plus Chemilumeniscent substrate (catalog 34580, Thermo Fisher Scientific). Membranes were then imaged on a Bio-Rad imager and analyzed using ImageLab software. The uncropped blots are available in Supplemental Figure 14.

### Basic processing of 16S amplicon sequencing data.

Forward and reverse reads were merged using PEAR, version 0.9.11. Merged reads were trimmed with cutadapt, version 4.4, to remove ambiguous nucleotides, primer sequences, and trimmed on the basis of a quality threshold of *P* = 0.01. Reads that were less than 225 bp after trimming were discarded. Chimeric sequences were identified and removed with VSEARCH, version 2.25.0, using the UCHIME algorithm with a comparison to Silva, version 138.1, reference sequence. Amplicon sequence variants (ASVs) were identified using DADA2, version 1.30.0. The representative sequences for each ASV were then annotated taxonomically using the Naive Bayesian classifier included in DADA2 with the Silva, version 138.1, training set.

### Differential analysis of Akkermansia.

Differential analyses of taxa as compared with the site were performed using the software package edgeR on raw sequence counts. Prior to analysis, the data were filtered to remove any sequences that were annotated as chloroplast or mitochondria in origin as well as removing taxa that accounted for less than 100 total counts and were present in less than 30% of the sample. Data were normalized as counts per million. Normalized data were then fit using a negative binomial generalized linear model using experimental covariates, and statistical tests were performed using a likelihood ratio test. Adjusted *P* values (*q* values) were calculated using the BH FDR correction. Significant taxa were determined on the basis of an FDR threshold of 5% (0.05). The data from this differential analysis are from Chandra et al. (9).

α*-Diversity analyses*. Shannon indices and richness (i.e., species number) were calculated with default parameters in R using the vegan library (https://vegandevs.github.io/vegan/). Prior to analysis, the data were rarefied to a depth of 10,000 counts per sample. The resulting Shannon indices were then modeled with the sample covariates using a generalized linear model (GLM) assuming a Gaussian distribution. Significance of the model (ANOVA) was tested using the *F* test. Post hoc tests were performed using Kruskal-Wallis test. Plots were generated in R using the ggplot2 library (https://ggplot2.tidyverse.org/).

### β-Diversity/dissimilarity analyses.

Bray-Curtis indices were calculated with default parameters in R using the vegan library. Prior to analysis, the normalized data were square-root-transformed. The resulting dissimilarity indices were modeled and tested for significance with the sample covariates using the ANOSIM test. Plots were generated in R using the ggplot2 library.

### Statistics.

Statistical analysis was performed using GraphPad Prism 9 software for all studies (Figures 1–3, 6 and 7, and Supplemental Figures 1–4, 7, and 10–12) except the RNA-Seq studies. With the exception of the RNA-Seq studies, comparisons between 2 groups were performed using 2-tailed, unpaired Student’s *t* test (Figures 1–3, and 6, and Supplemental Figures 1–4, 7, and 10–12). Multiple comparisons were performed using 1- or 2-way ANOVA (Figures 2 and 7, and Supplemental Figure 3). Pearson’s correlation coefficients and 2-tailed *P* values were calculated using GraphPad Prism 9 (GraphPad Software) with a CI of 95%. Spearman’s correlation coefficients and 2-tailed *P* values were calculated for Figure 2I. Data are expressed as the mean ± SD. Statistical significance was defined as a *P* value less than 0.05, with significance levels indicated as follows: *P < 0.05, **P < 0.01, ***P < 0.001, ****P < 0.0001. NS denotes not significant (*P* > 0.05).

### Study approval.

All experimental procedures for these mice were approved by the IACUC of Northwestern University and the University of Chicago.

### Data availability.

All data represented in the study are available in the Supporting Data Values file. snRNA-Seq and TRAP-Seq files have been deposited in the Gene Expression Omnibus (GEO) database (GSE295458 and GSE295459). GC-MS raw files can be shared upon request.

## Author contributions

SC and RV conceived the study. SJS, MP, DG, and SSS provided guidance as the study evolved. SC, JP, NKS, EAW, HBD, IQW, MAS, AR, ZC, AS, XZ, LC, and KRS performed the experiments. JTW and NP helped perform bioinformatics analysis on the snRNA-Seq data. SC and RV wrote the manuscript. DG and SSS helped edit the manuscript. All authors read and approved the final manuscript.

## Supplementary Material

Supplemental data

Unedited blot and gel images

Supplemental table 1

Supplemental table 2

Supplemental table 3

Supplemental table 4

Supplemental table 5

Supporting data values

## Version 1 05/13/2025

In-Press Preview

## Version 2 07/01/2025

Electronic publication

## References

[1] Long JM, Holtzman DM (2019). Alzheimer disease: an update on pathobiology and treatment strategies. Cell.

[2] Vogt NM (2017). Gut microbiome alterations in Alzheimer’s disease. Sci Rep.

[3] Minter MR (2016). Antibiotic-induced perturbations in gut microbial diversity influences neuro-inflammation and amyloidosis in a murine model of Alzheimer’s disease. Sci Rep.

[4] Minter MR (2017). Antibiotic-induced perturbations in microbial diversity during post-natal development alters amyloid pathology in an aged APP_SWE_/PS1_ΔE9_ murine model of Alzheimer’s disease. Sci Rep.

[5] Dodiya HB (2019). Sex-specific effects of microbiome perturbations on cerebral Aβ amyloidosis and microglia phenotypes. J Exp Med.

[6] Dodiya HB (2021). Gut microbiota-driven brain Aβ amyloidosis in mice requires microglia. J Exp Med.

[7] Seo DO (2023). ApoE isoform- and microbiota-dependent progression of neurodegeneration in a mouse model of tauopathy. Science.

[8] Chandra S, Vassar R (2024). The role of the gut microbiome in the regulation of astrocytes in Alzheimer’s disease. Neurotherapeutics.

[9] Chandra S (2023). The gut microbiome regulates astrocyte reaction to Aβ amyloidosis through microglial dependent and independent mechanisms. Mol Neurodegener.

[10] Liddelow SA (2017). Neurotoxic reactive astrocytes are induced by activated microglia. Nature.

[11] Chandra S, Vassar RJ (2024). Gut microbiome-derived metabolites in Alzheimer’s disease: Regulation of immunity and potential for therapeutics. Immunol Rev.

[12] Cani PD (2022). Akkermansia muciniphila: paradigm for next-generation beneficial microorganisms. Nat Rev Gastroenterol Hepatol.

[13] Bosch ME (2024). Sodium oligomannate alters gut microbiota, reduces cerebral amyloidosis and reactive microglia in a sex-specific manner. Mol Neurodegener.

[14] Heiman M (2014). Cell type-specific mRNA purification by translating ribosome affinity purification (TRAP). Nat Protoc.

[15] Erny D (2015). Host microbiota constantly control maturation and function of microglia in the CNS. Nat Neurosci.

[16] Dodiya HB (2020). Synergistic depletion of gut microbial consortia, but not individual antibiotics, reduces amyloidosis in APPPS1-21 Alzheimer’s transgenic mice. Sci Rep.

[17] Annunziato F (2012). Defining the human T helper 17 cell phenotype. Trends Immunol.

[18] Ramakrishnan A (2024). Epigenetic dysregulation in Alzheimer’s disease peripheral immunity. Neuron.

[19] Gaublomme JT (2015). Single-cell genomics unveils critical regulators of Th17 cell pathogenicity. Cell.

[20] Mezö C (2020). Different effects of constitutive and induced microbiota modulation on microglia in a mouse model of Alzheimer’s disease. Acta Neuropathol Commun.

[21] Xie J (2023). Gut microbiota regulates blood-cerebrospinal fluid barrier function and Aβ pathology. EMBO J.

[22] Wu L (2021). Altered gut microbial metabolites in amnestic mild cognitive impairment and alzheimer’s disease: signals in host-microbe interplay. Nutrients.

[23] Zheng J (2019). Stable isotope labeling combined with liquid chromatography-tandem mass spectrometry for comprehensive analysis of short-chain fatty acids. Anal Chim Acta.

[24] Erny D (2021). Microbiota-derived acetate enables the metabolic fitness of the brain innate immune system during health and disease. Cell Metab.

[25] Sun C-Y (2023). T helper 17 (Th17) cell responses to the gut microbiota in human diseases. Biomed Pharmacother.

[26] Du HX (2022). Gut microflora modulates Th17/Treg cell differentiation in experimental autoimmune prostatitis via the short-chain fatty acid propionate. Front Immunol.

[27] Haghikia A (2015). Dietary fatty acids directly impact central nervous system autoimmunity via the small intestine. Immunity.

[28] Cao M (2023). IL-17A promotes the progression of Alzheimer’s disease in APP/PS1 mice. Immun Ageing.

[29] Lian H (2016). Astrocyte-microglia cross talk through complement activation modulates amyloid pathology in mouse models of Alzheimer’s disease. J Neurosci.

[30] Chandra S (2023). The gut microbiome in Alzheimer’s disease: what we know and what remains to be explored. Mol Neurodegener.

[31] Colombo AV (2021). Microbiota-derived short chain fatty acids modulate microglia and promote Aβ plaque deposition. Elife.

[32] Luczynski P (2016). Growing up in a bubble: using germ-free animals to assess the influence of the gut microbiota on brain and behavior. Int J Neuropsychopharmacol.

[33] Duscha A (2020). Propionic acid shapes the multiple sclerosis disease course by an immunomodulatory mechanism. Cell.

[34] Smith PM (2013). The microbial metabolites, short-chain fatty acids, regulate colonic Treg cell homeostasis. Science.

[35] Naik S (2015). Commensal-dendritic-cell interaction specifies a unique protective skin immune signature. Nature.

[36] Hofstetter HH (2005). Therapeutic efficacy of IL-17 neutralization in murine experimental autoimmune encephalomyelitis. Cell Immunol.

[37] Burrell BE (2008). CD8^+^ Th17 mediate costimulation blockade-resistant allograft rejection in T-bet-deficient mice. J Immunol.

[38] Haak BW (2018). Impact of gut colonization with butyrate-producing microbiota on respiratory viral infection following allo-HCT. Blood.

[39] Martin M (2011). Cutadapt removes adapter sequences from high-throughput sequencing reads. EMBnet J.

[40] Putri G (2022). Analysing high-throughput sequencing data in Python with HTSeq 2.0. Bioinformatics.

