# Supplementary material for: The gut microbiome controls reactive astrocytosis during Aβ amyloidosis via propionate-mediated regulation of IL-17
Source: J Clin Invest. 2025 May 13;135(13):e180826. doi: 10.1172/JCI180826 (PMC12208551; doi:10.1172/JCI180826)
Supplement: Supplemental data [file jci-135-180826-s009.pdf]

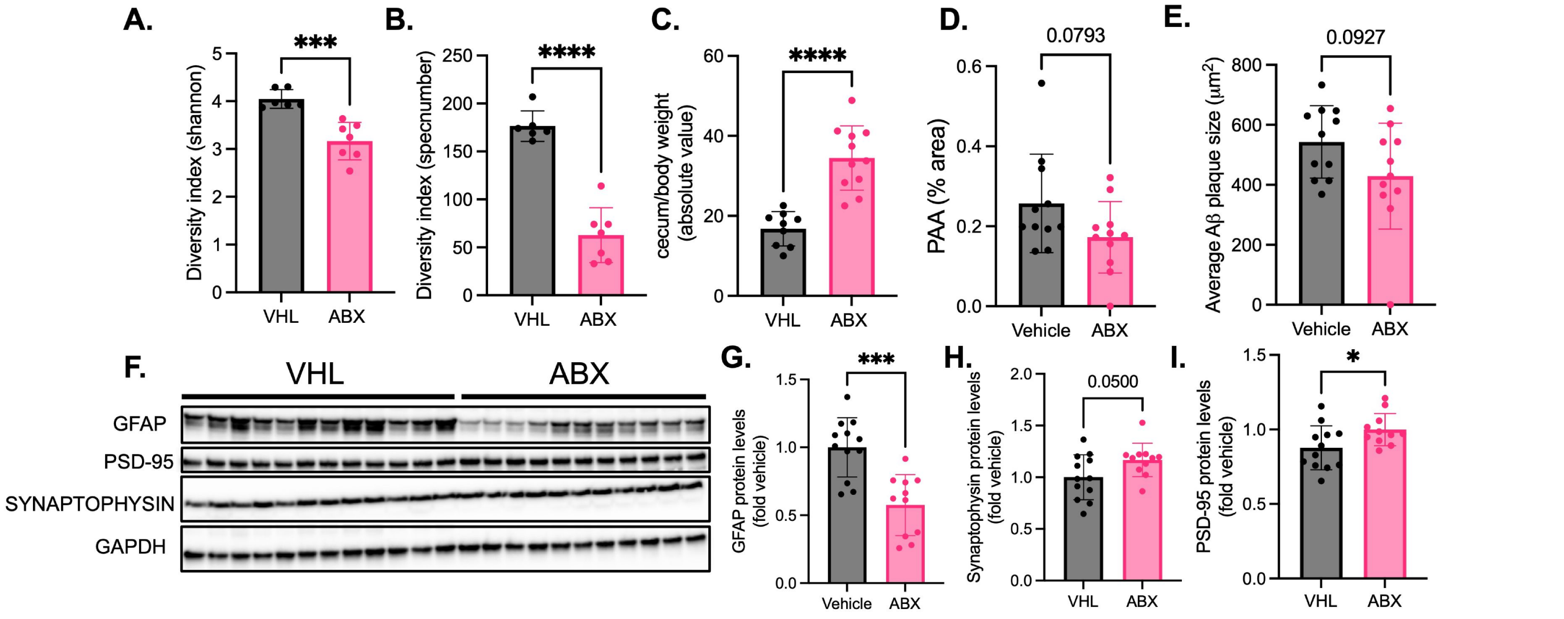

Supplemental Figure 1: Antibiotic-mediated changes in microbial diversity and synaptic health in APPPS1-21 male mice.

(A) Shannon diversity index in vehicle (VHL) and antibiotic (ABX) treated APPPS1-21 male mice at amplicon sequence variant (ASV) level. (B) Specnumber diversity index in VHL and ABX treated APPPS1-21 male mice at ASV level. (C) Cecum/body weight measurements in ABX-treated APPPS1-21 male mice. (D) Cortical plaque-associated astrocyte (PAA) percent area in ABX-treated APPPS1-21 male mice. (E) Average Aβ plaque size in ABX-treated APPPS1-21 male mice. (F) Immunoblot of GFAP, PSD-95, synaptophysin, and GAPDH loading control and quantification (G-I) in cortical lysates from VHL and ABX treated male APPPS1-21 mice. Data expressed as mean ± standard deviation. N = 6-12/group. Statistics calculated using two-tailed unpaired student's t-tests. * denotes a p-value ≤ 0.05, ** indicates p-value ≤ 0.01, *** indicates p-value ≤ 0.001, and **** indicates a p-value of ≤ 0.0001.

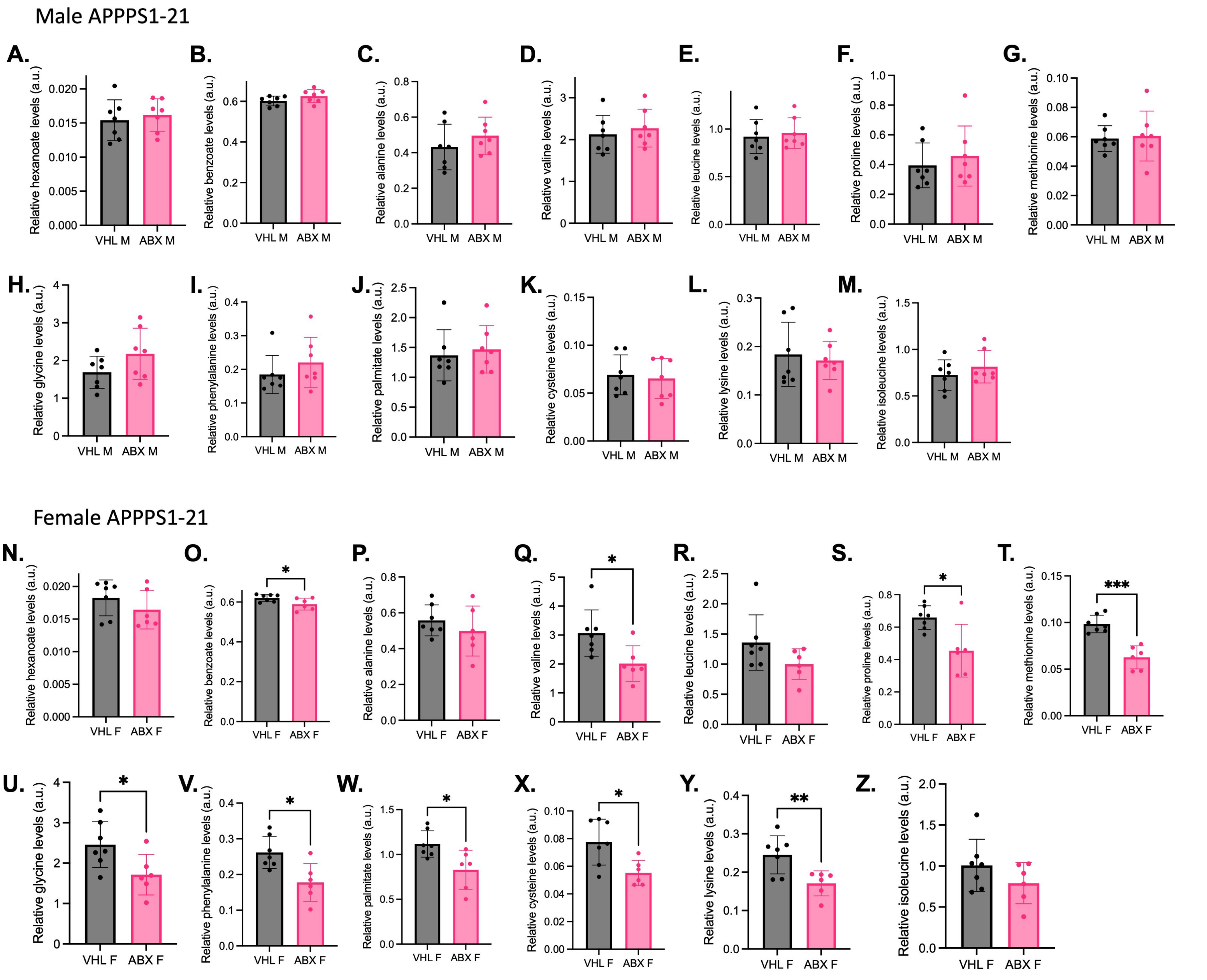

Supplemental Figure 2: Antibiotic treatment alters levels of gut-derived metabolites in the plasma of APPPS1-21 male and female mice.

A-M: Levels of gut-derived metabolites measured in the plasma in vehicle and abx treated APPPS1-21 male mice.

N-Z: Levels of gut-derived metabolites measured in the plasma in vehicle and abx treated APPPS1-21 female mice.

Data expressed as mean ± standard deviation. N = 6-7/group. Statistics calculated using two-tailed unpaired student's t-tests. * denotes a p-value ≤ 0.05, ** indicates p-value ≤ 0.01, *** indicates p-value ≤ 0.001, and **** indicates a p-value of ≤ 0.0001.

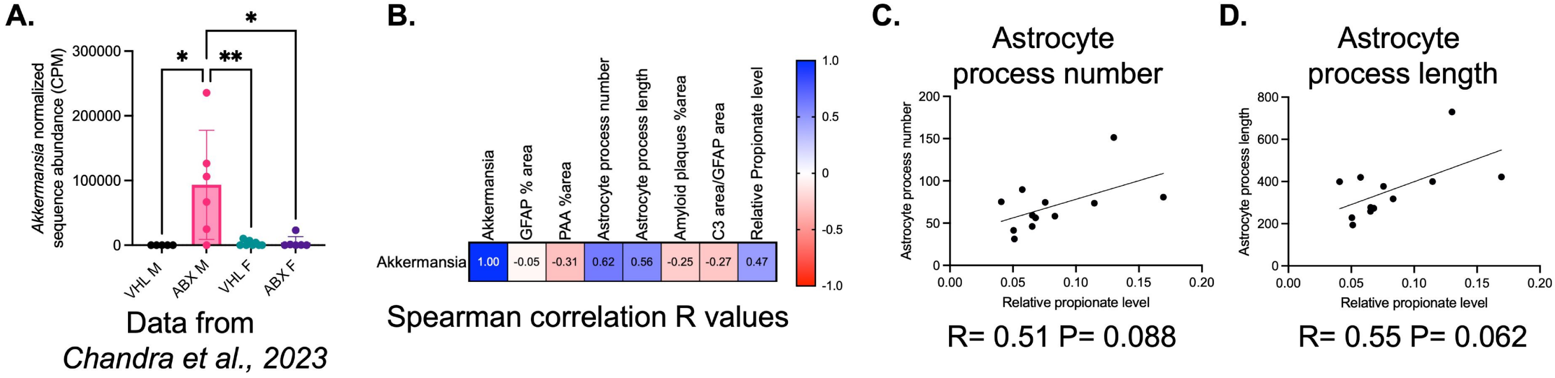

Supplemental Figure 3: Increase in Akkermansia following antibiotic treatment in male APPPS1-21 mice. (A) Quantification of *Akkermanisa* normalized sequence abundance between VHL and ABX treated APPPS1-21 male (M) and female (F) mice calculated from original data reported in Chandra et al., 2023. The same abx-treated mice were used for measurement of gut-derived metabolites in the plasma shown in Fig. 2. (B) Spearman's matrix of correlational R values for correlations between *Akkermanisa* levels and GFAP+ astrocytes, PAAs, astrocyte process number/length, C3 area/GFAP area, and propionate levels. Pearson's correlation analysis between (C) astrocyte process number and (D) astrocyte process length and plasma propionate levels in VHL and ABX treated male APPPS1-21 mice. Data expressed as mean ± standard deviation. N = 6-7/group. Statistics calculated using two-way ANOVA. * denotes a p-value ≤ 0.05, ** indicates p-value ≤ 0.01, *** indicates p-value ≤ 0.001, and **** indicates a p-value of ≤ 0.0001.

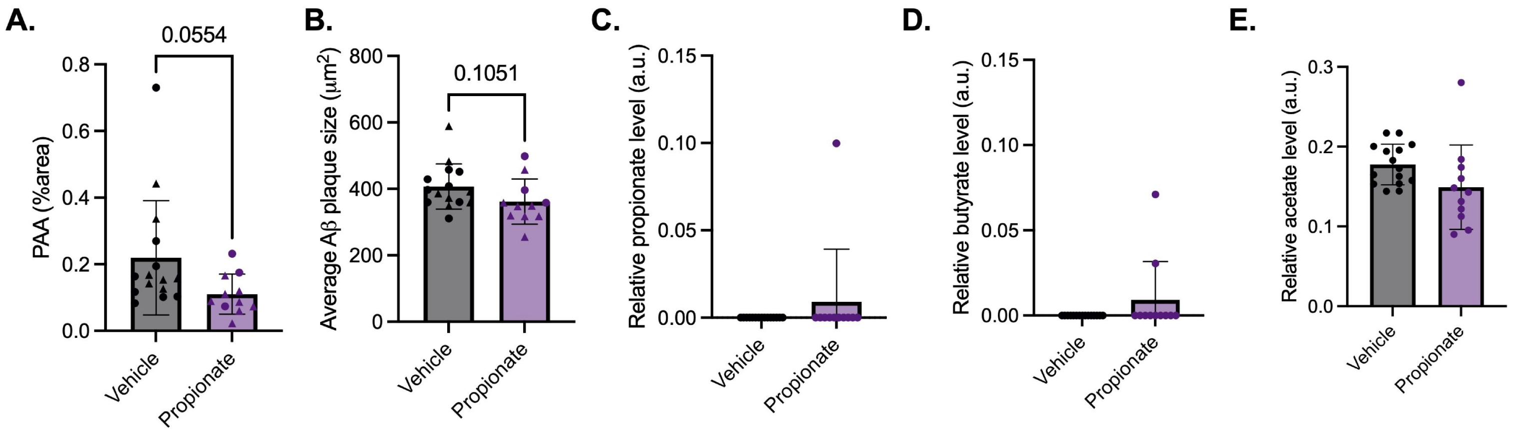

Supplemental Figure 4: Exogenous propionate treatment may reduce plaque-associated astrocytes and Aβ plaque size in APPPS1-21 mice but does not reach appreciable levels in the cortex.
Quantification of cortical (A) GFAP+ plaque-associated astrocyte (PAA) percent area and (B) Aβ plaque size in VHL and PROP treated APPPS1-21 male and female mice. Relative (C) propionate, (D) butyrate, and (E) acetate levels in hemicortex of VHL and PROP treated APPPS1-21 mice. Data expressed as mean ± standard deviation. N = 11-15/group. Statistics calculated using two-tailed unpaired student's t-tests. 4 sections used per animal. Males denoted by triangles and females denoted by circles.

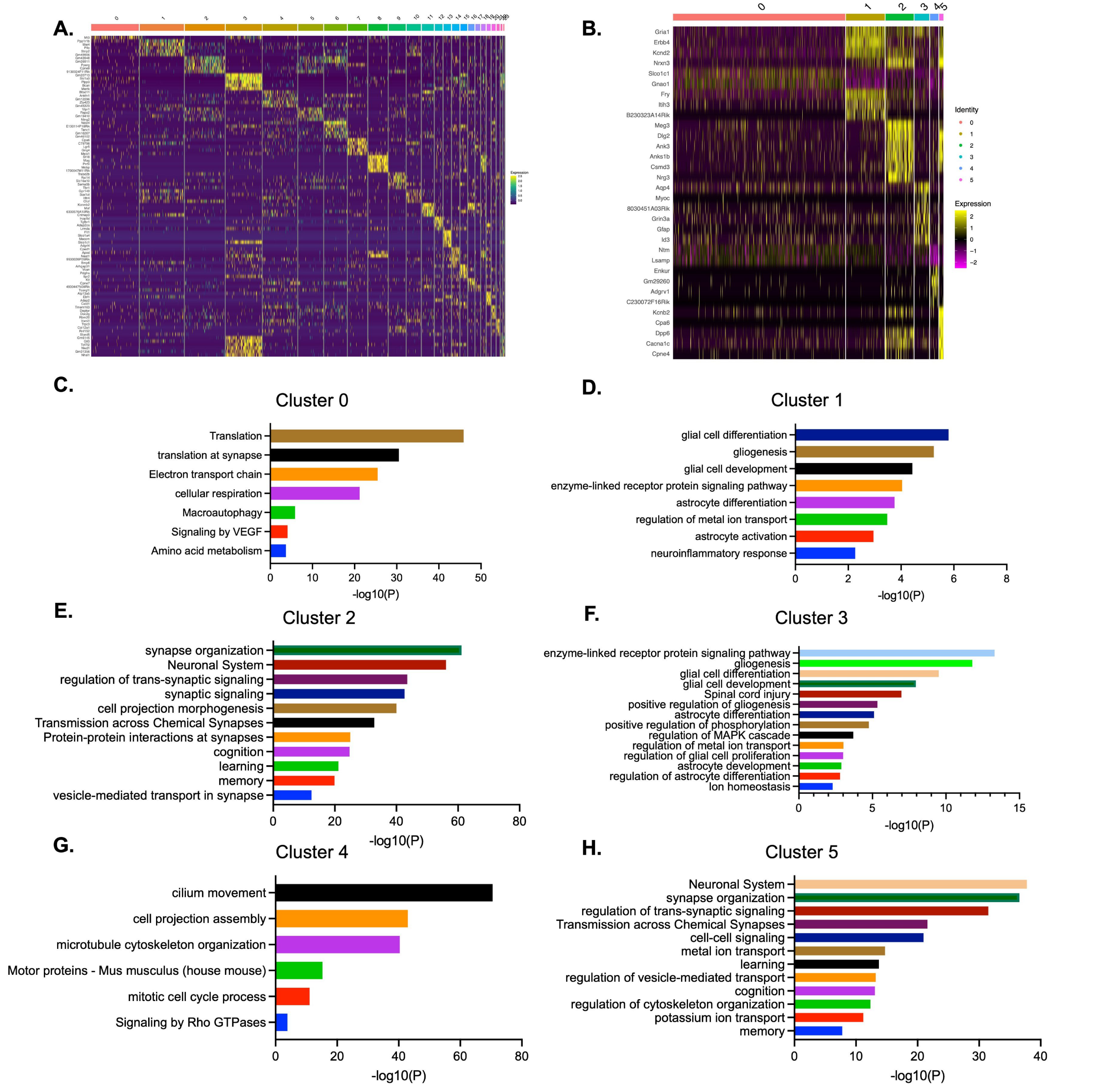

Supplemental Figure 5: Heatmaps of enriched gene markers and pathway analyses of astrocyte subclusters
(A) Heatmap of enriched gene markers in (A) UMAP containing all cells and (B) astrocyte subcluster UMAP. Astrocyte subclusters 0-5 (C-H) enriched pathways. Pathways determined using Metascape.

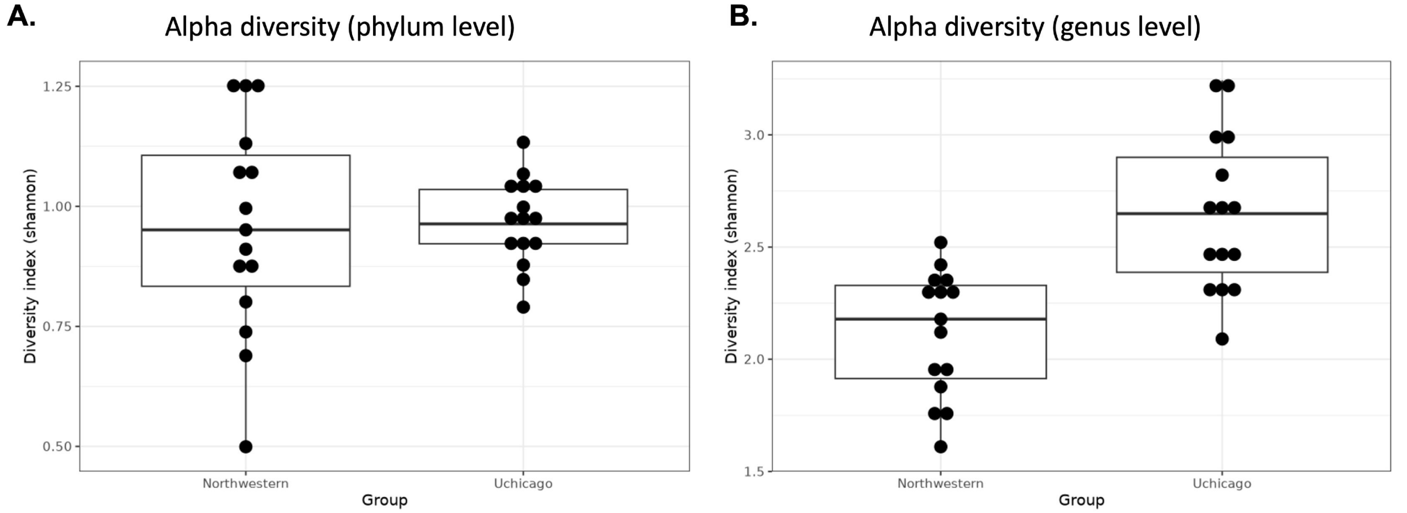

Kruskal-Wallis p-value= 0.917411

Kruskal-Wallis p-value= 0.000333412

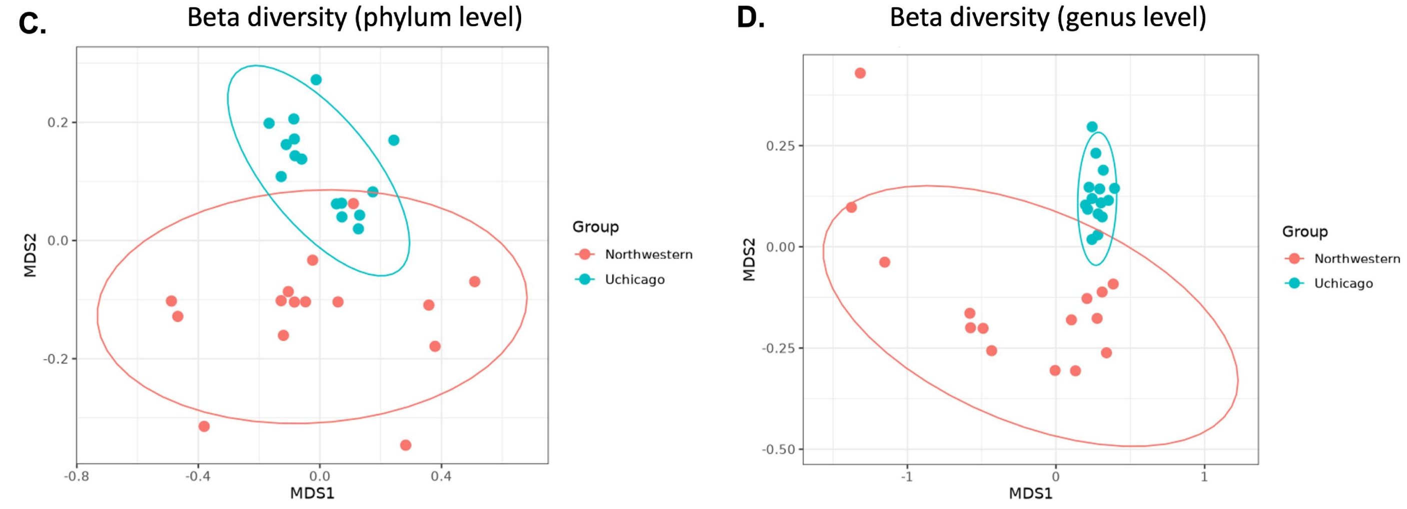

High level ANOSIM

Factor	R	P-value
Location	0.335	0.001

High level ANOSIM

Factor	R	P-value
Location	0.489	0.001

Supplemental Figure 6: Facility-dependent changes in fecal microbiome alpha and beta diversity. Alpha diversity analysis comparing 9-week-old male APPPS1-21 fecal microbiome using 16S rRNA sequencing between Northwestern University and University of Chicago at (A) phylum level and (B) genus level. Beta diversity analysis comparing fecal microbiome using 16S rRNA sequencing between Northwestern University and University of Chicago at (C) phylum level and (D) genus level. The significance of the alpha-diversity model (ANOVA) was tested using the F test. Post-hoc tests were performed using Kruskal-Wallis test. Beta dissimilarity indices were modelled and tested for significance with the sample covariates using the ANOSIM test.

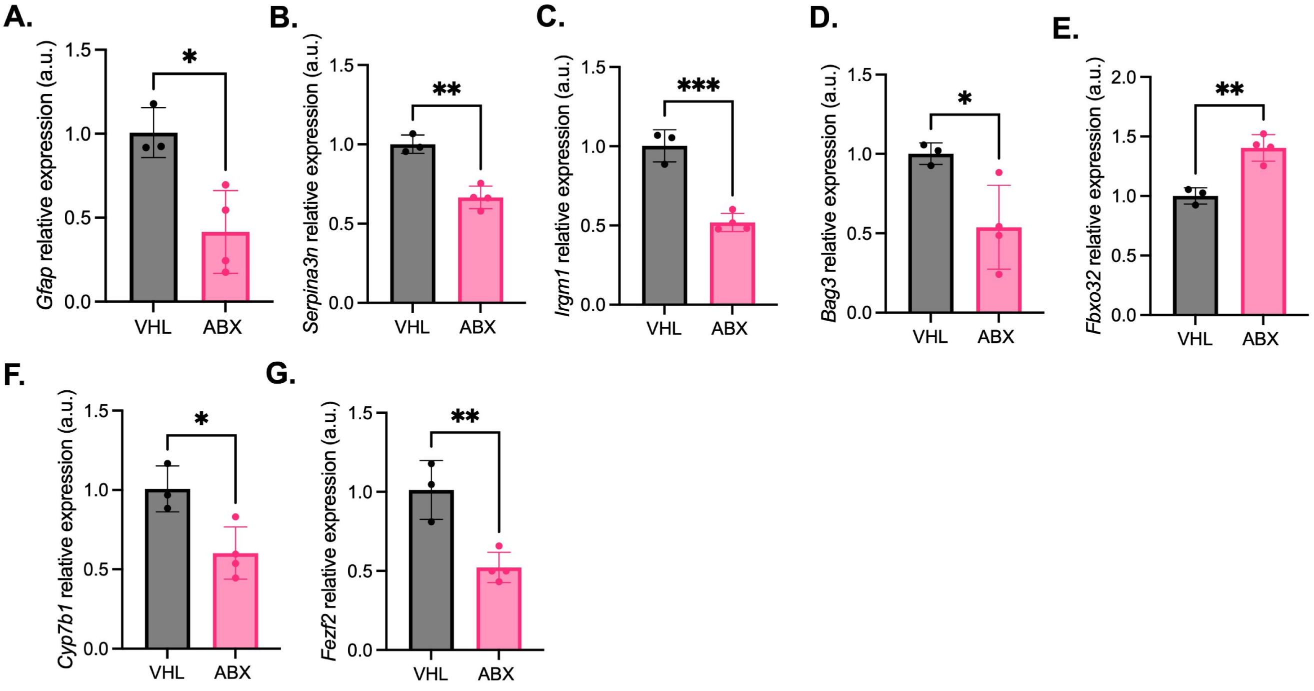

Supplemental Figure 7: qPCR Validation of TRAPseq gene changes in antibiotic-treated male APPPS1-21 mice.

Relative gene expression as determined by qRCR of (A) Gfap, (B) Serpina3n, and (C) Irgm1, (D) Bag3, (E) Fbxo32, (F) Cyp7b1, (G) Fezf2 in male APPPS1-21 VHL and ABX treated mice. Data expressed as mean ± standard deviation, N = 3-4/group, a.u. = arbitrary units. Statistics calculated using two-tailed unpaired student's t-tests. * denotes a p-value ≤ 0.05, ** indicates p-value ≤ 0.01, and *** indicates p-value ≤ 0.001.

A.

Genes significantly regulated in the same direction between ABX and Propionate in TRAPseq

Genes	Regulation
Gfap	Downregulated
Irgm1	Downregulated
Fezf2	Downregulated
Pinx1	Downregulated
Sall2	Downregulated
Cables1	Downregulated
Map3k19	Downregulated
Tead3	Downregulated
Safb	Downregulated
Ncoa5	Downregulated
Vsir	Downregulated
Fxr2	Downregulated
Bag3	Downregulated
Soga3	Downregulated
Gm973	Downregulated
Sart1	Downregulated
Scrn1	Downregulated
Cyp7b1	Downregulated
Fbxo32	Upregulated
Szt2	Upregulated
Cdc14b	Upregulated
Taf4b	Upregulated

B.

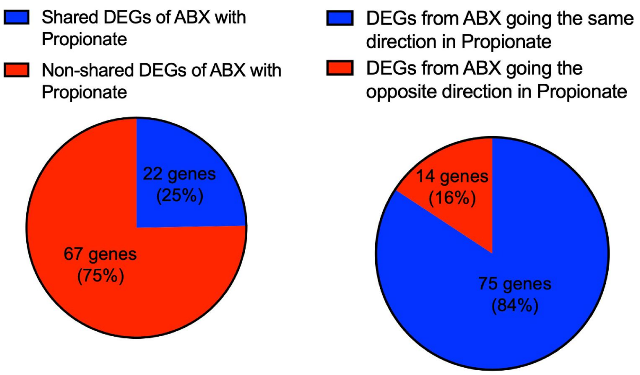

C.

Molecular pathways in common between ABX and Propionate in TRAPseq

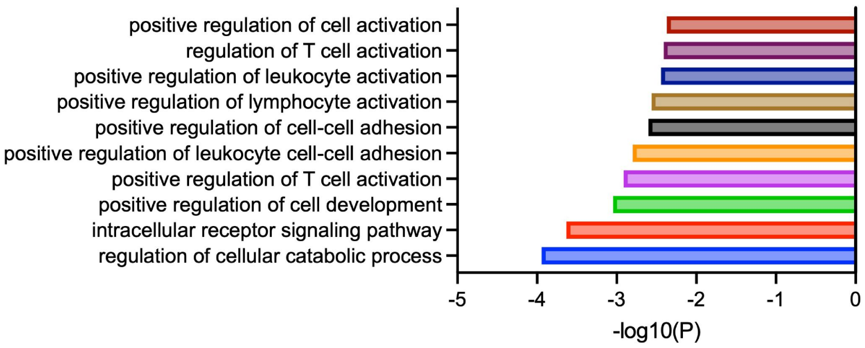

Supplemental Figure 8: Genes and pathways shared between antibiotic and propionate treated APPPS1-21 mice.
(A) Shared DEGs as determined by TRAPseq between ABX and PROP treated APPPS1-21 mice with the same direction of Log2FC. (B) Pie chart of ABX and PROP shared DEGs and shared Log2FC directions. (C) Pathway analysis depicting up and down-regulated molecular pathways using shared ABX and PROP DEGs.

Th17 gating strategy

A.

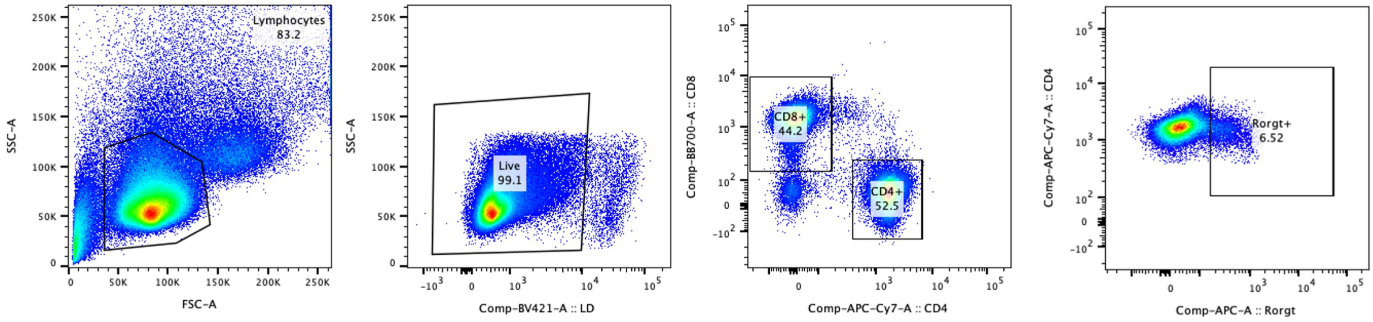

Supplemental Figure 9: Flow cytometry gating strategy of Rorγt+ CD4+ Th17 cells.
(A) Total lymphocytes were first gated into live cells, then CD4+ cells, and finally Rorγt+ cells.

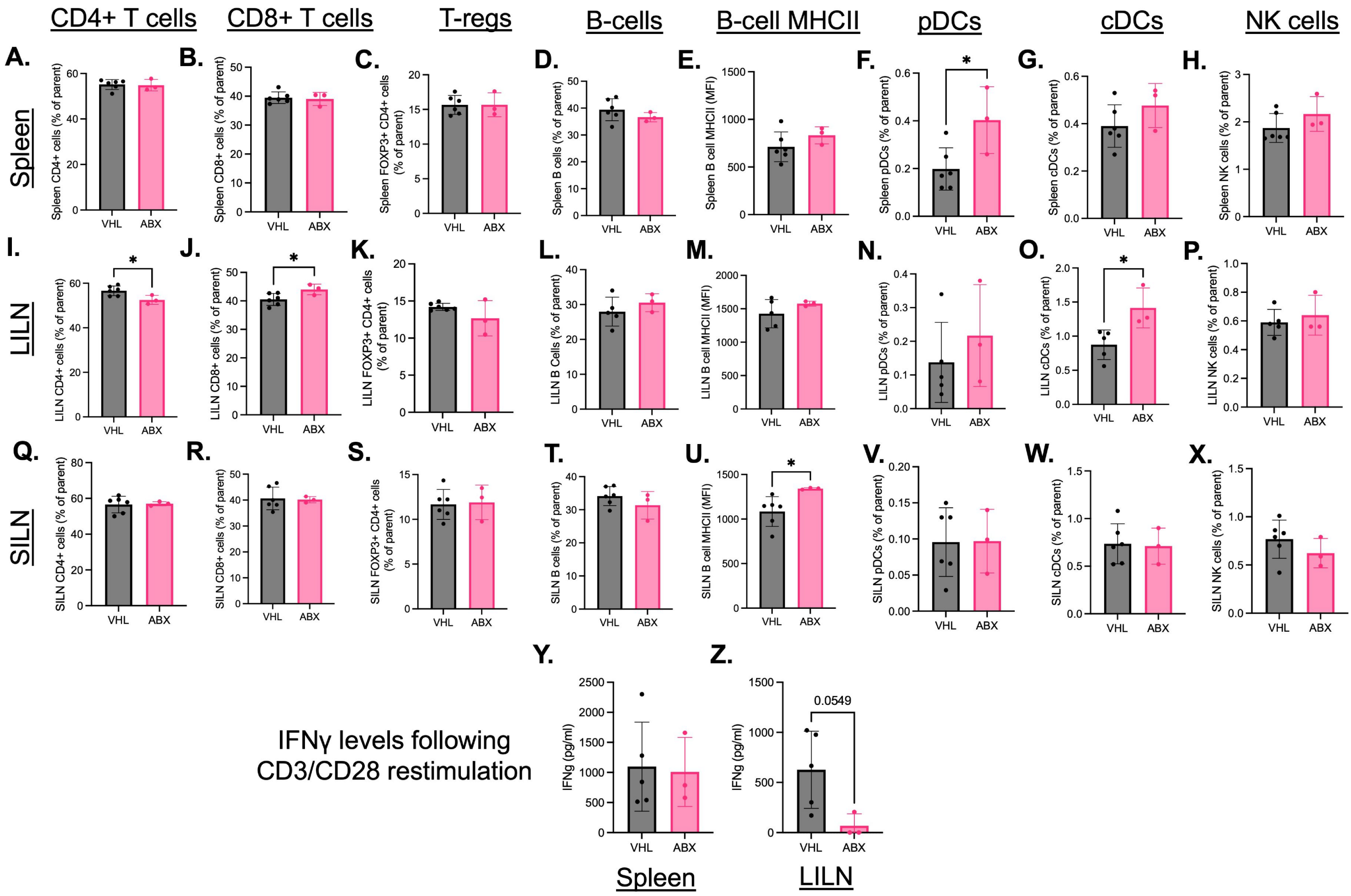

Supplemental Figure 10: Changes in peripheral immune cells in spleen, large intestine, and small intestine following antibiotic treatment in APPPS1-21 male mice.
Changes in flow cytometry percentages of CD4+ T-cells, CD8+ T-cells, Tregs, B cells, B cell MHCII, pDCs, cDCs, NK cells in the (A-H) spleen, (I-P) LILN, and (Q-X) SILN of antibiotic (ABX) treated APPPS1-21 male mice compared to vehicle (VHL) controls. IFNγ protein levels measured by ELISA in the media from cells plated from the (Y) spleen and (Z) LILN of VHL and ABX treated mice after CD3/CD28 bead restimulation. Data expressed as mean ± standard deviation. N = 3-6/group. Statistics calculated using two-tailed unpaired student's t-tests. * denotes a p-value ≤ 0.05, ** indicates p-value ≤ 0.01, *** indicates p-value ≤ 0.001, and **** indicates a p-value of ≤ 0.0001.

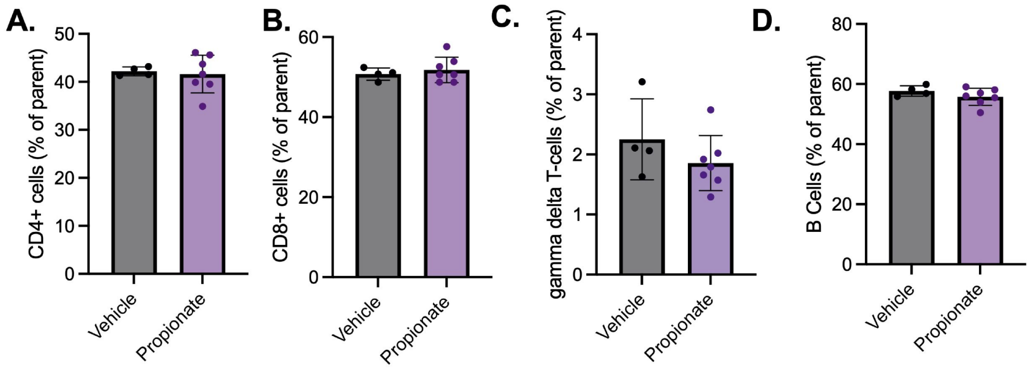

Supplemental Figure 11: Changes in peripheral immune cells in the plasma following propionate treatment in APPPS1-21 male mice.

Changes in (A) CD4+, (B) CD8+, (C) gamma delta T-cells, and (D) B cells in the plasma of propionate treated APPPS1-21 male mice compared to vehicle (VHL) controls by flow cytometry. Data expressed as mean ± standard deviation. N = 4-7/group. Statistics calculated using two-tailed unpaired student's t-tests.

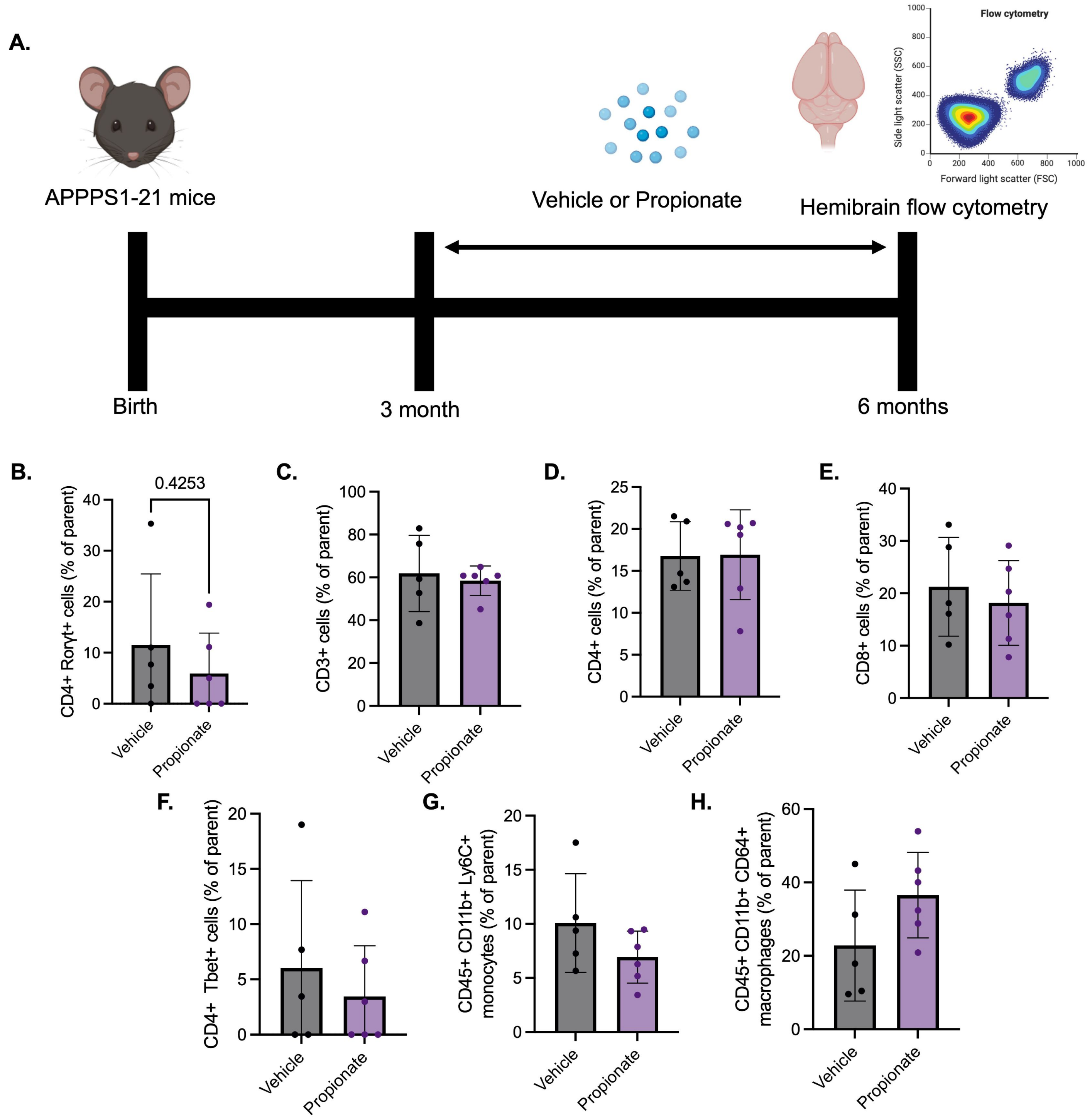

Supplemental Figure 12: No changes in immune cell populations in the brain following propionate treatment. (A) Schematic depicting experimental paradigm. (B-H) Changes in flow cytometry percentages of Rorγt+ CD4+ Th17 cells, CD3+ T-cells, CD4+ T-cells, CD8+ T-cells, Tbet+ CD4+ Th1 cells, monocytes, and macrophages in the hemibrains of propionate treated APPPS1-21 male mice compared to vehicle (VHL) controls. Data expressed as mean ± standard deviation. N = 5-6/group. Statistics calculated using two-tailed unpaired student's t-tests.

Adapted from *Ramakrishnan et al., Neuron, 2024*

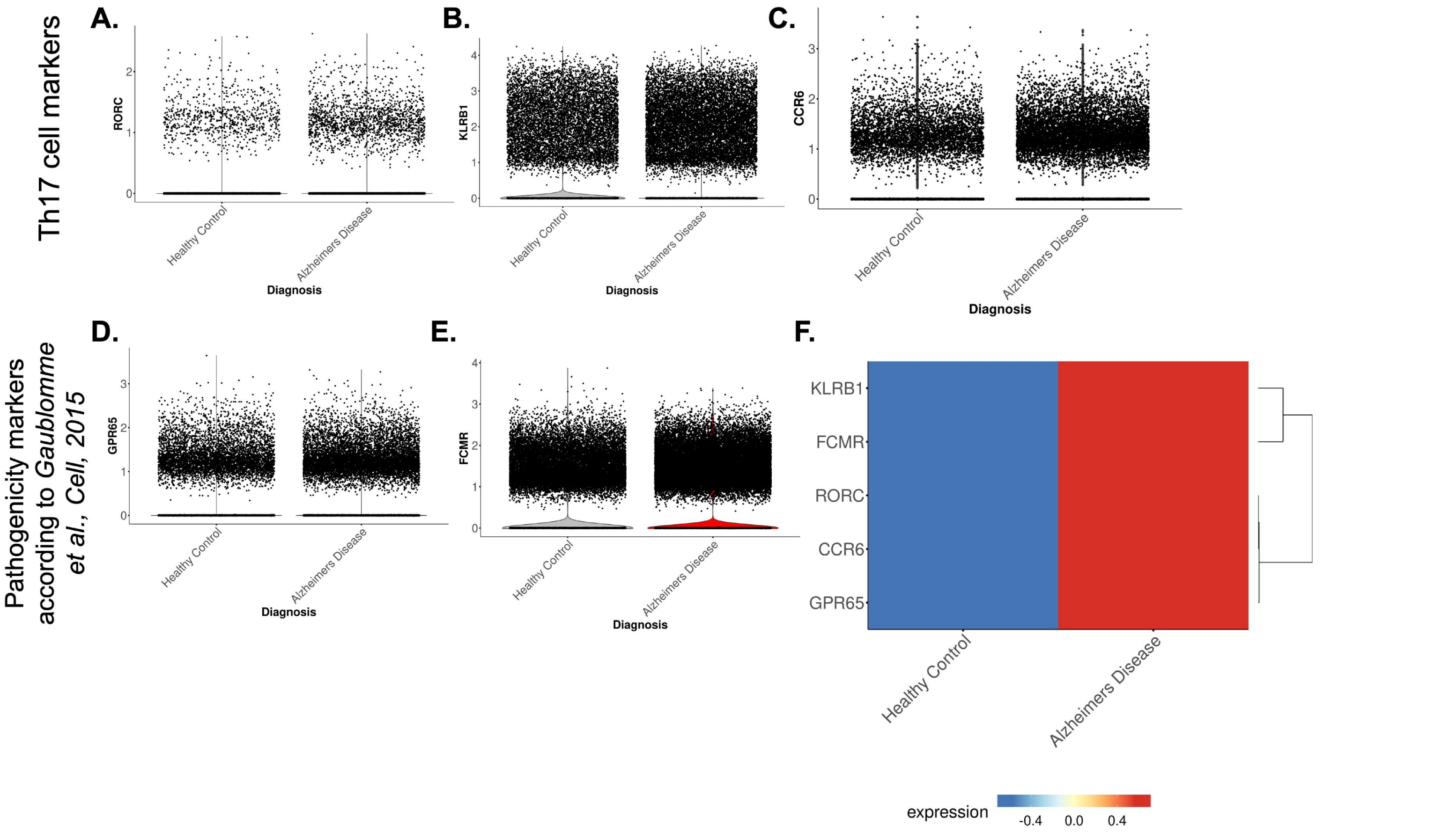

Supplemental Figure 13: Increases in circulating pathogenic Th17 cells in human AD.
(A) RORC, (B) KLRB1, (C) CCR6, (D) GPR65, and (E) FCMR expression in human AD PBMCs compared to healthy controls in Ramakrishnan et al., Neuron, 2024 dataset. (F) Heatmap representing relative gene expression of Th17 and pathogenic Th17 markers in Ramakrishnan et al., Neuron, 2024 dataset.

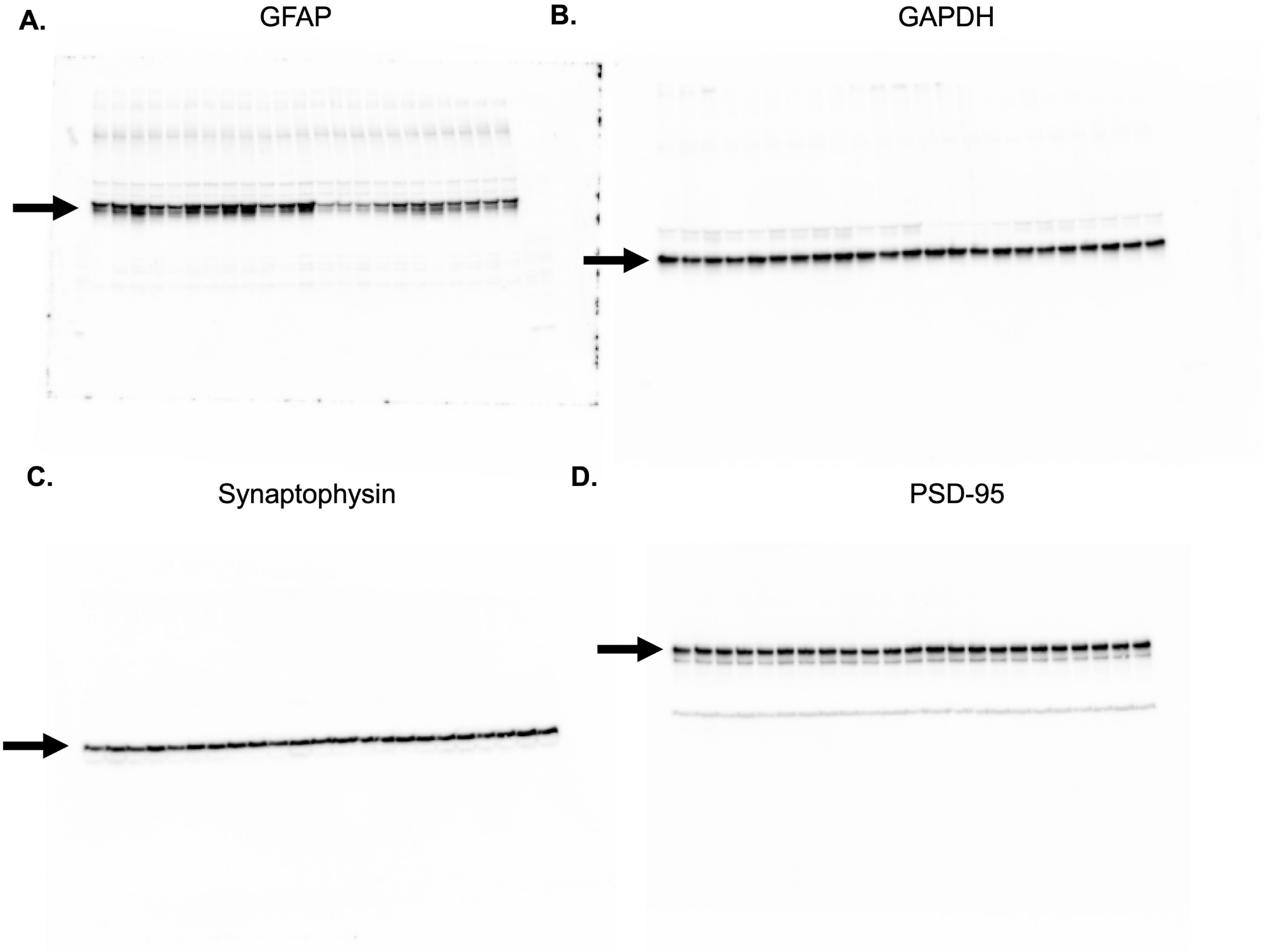

Supplemental Figure 14: Uncropped immunoblots represented in Supplemental Figure 1.
Uncropped (A) GFAP, (B) GAPDH, (C) Synaptophysin, and (D) PSD-95 blots represented in Supplemental Figure 1. Arrows indicate which bands were used in the cropped images.
