# Supplementary material for: The gut microbiome controls reactive astrocytosis during Aβ amyloidosis via propionate-mediated regulation of IL-17
Source: J Clin Invest. 2025 May 13;135(13):e180826. doi: 10.1172/JCI180826 (PMC12208551; doi:10.1172/JCI180826)
Supplement: Unedited blot and gel images [file jci-135-180826-s010.pdf]

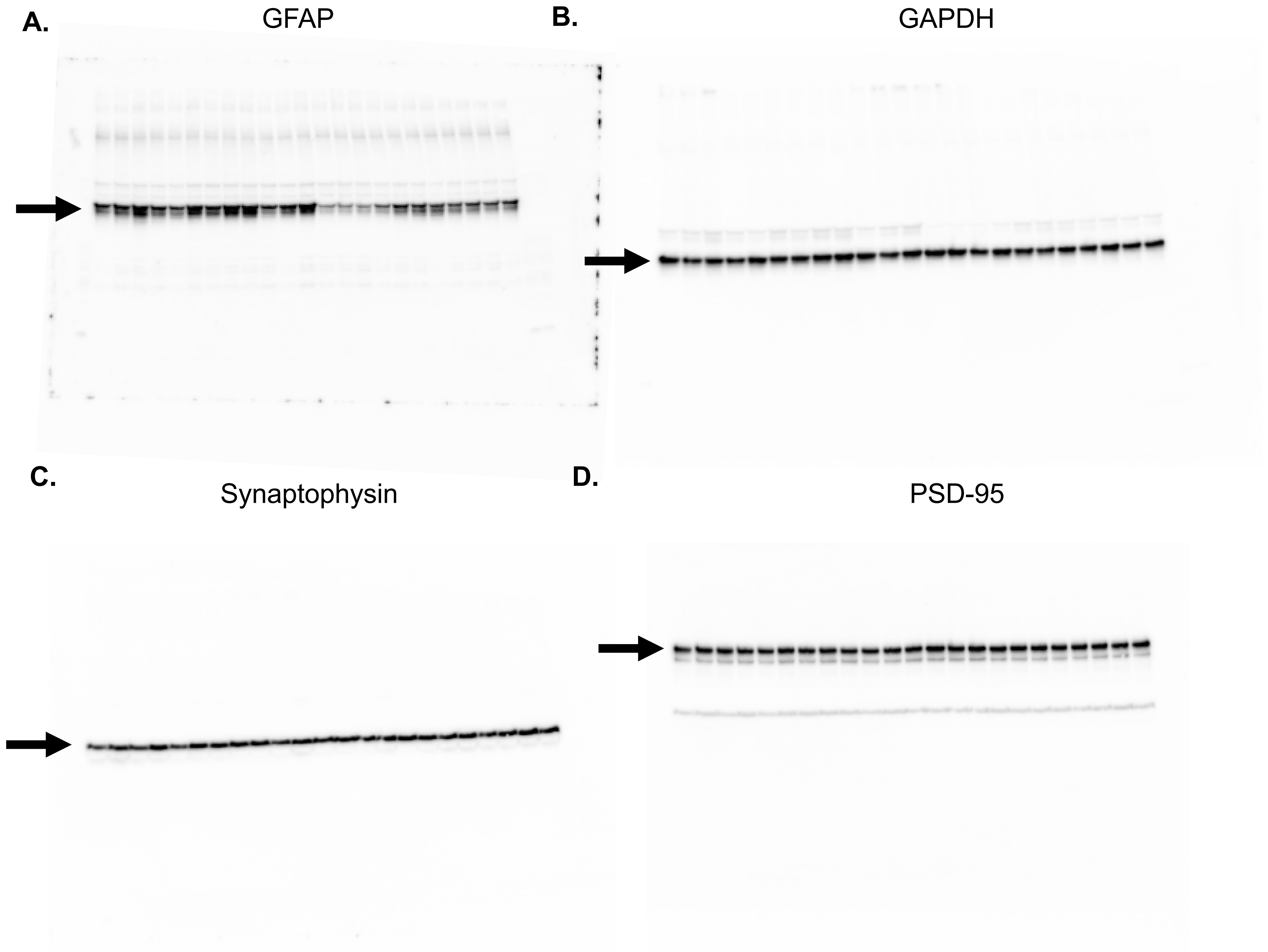

Supplemental Figure 14: Uncropped immunoblots represented in Supplemental Figure 1.
Uncropped (A) GFAP, (B) GAPDH, (C) Synaptophysin, and (D) PSD-95 blots represented in Supplemental Figure 1. Arrows indicate which bands were used in the cropped images.
